# A global dataset of CO_2_ emissions and ancillary data related to emissions for 343 cities

**DOI:** 10.1038/sdata.2018.280

**Published:** 2019-01-15

**Authors:** Cathy Nangini, Anna Peregon, Philippe Ciais, Ulf Weddige, Felix Vogel, Jun Wang, François-Marie Bréon, Simeran Bachra, Yilong Wang, Kevin Gurney, Yoshiki Yamagata, Kyra Appleby, Sara Telahoun, Josep G. Canadell, Arnulf Grübler, Shobhakar Dhakal, Felix Creutzig

**Affiliations:** 1 Laboratoire des Sciences du Climat et de l’Environnement CEA CNRS UVSQ Institut Pierre-Simon Laplace, Gif-sur-Yvette, France; 2 Mercator Research Institute on Global Commons and Climate Change, Berlin, Germany; 3 Climate Research Division, Environment and Climate Change Canada, Toronto, Canada; 4 Peking University Shenzhen Graduate School, Key Laboratory for Human and Environmental Science and Technology, Shenzhen, China; 5 CDP Worldwide, London, England; 6 Arizona State University, Arizona, USA; 7 National Institute for Environmental Studies, Tsukuba, Japan; 8 Global Carbon Project, CSIRO Oceans and Atmosphere, Canberra, Australia; 9 International Institute for Applied Systems Analysis, Laxenburg, Austria; 10 Department of Energy, Environment and Climate Change, Asian Institute of Technology, Klong Luang, Pathumthani, Thailand; 11 Sustainability Economics of Human Settlements, Technical University Berlin, Berlin Germany

**Keywords:** Carbon and energy, Environmental economics, Climate sciences, Environmental chemistry

## Abstract

We present a global dataset of anthropogenic carbon dioxide (CO_2_) emissions for 343 cities. The dataset builds upon data from CDP (187 cities, few in developing countries), the Bonn Center for Local Climate Action and Reporting (73 cities, mainly in developing countries), and data collected by Peking University (83 cities in China). The CDP data being self-reported by cities, we applied quality control procedures, documented the type of emissions and reporting method used, and made a correction to separate CO_2_ emissions from those of other greenhouse gases. Further, a set of ancillary data that have a direct or potentially indirect impact on CO_2_ emissions were collected from other datasets (e.g. socio-economic and traffic indices) or calculated (climate indices, urban area expansion), then combined with the emission data. We applied several quality controls and validation comparisons with independent datasets. The dataset presented here is not intended to be comprehensive or a representative sample of cities in general, as the choice of cities is based on self-reporting not a designed sampling procedure.

## Background & Summary

Cities are hotspots of the global carbon cycle, with considerable fossil fuel and cement CO_2_ emissions from the provision (9.2 GtCO_2_) and use (9.6 GtCO_2_) of urban infrastructure^1^. Cities concentrate population, energy use^2^ and economic output, hence are important focal points for investigating emission drivers and mitigation options, prompting across-city comparative analyses of greenhouse gases (GHG) emissions^3,4^ and energy use^2^.

To understand city emission drivers in different global regions, not only city-scale CO_2_ emissions estimates are needed from local inventories, but also underlying ancillary socio-economic data. Scope-1 emissions cover GHGs emitted in the city territory, including emissions from grid-supplied energy produced within cities^5^. Scope-2 emissions include grid-supplied energy used by cities and produced by power plants outside the city boundary. The distinction about power plants emissions included in Scope-1 is not always precise in city-reported data. Scope-1 GHG emissions of cities^5^ include transport, industrial, waste and local power plants emissions, and allow for a more direct comparison between cities, as uncertain additional assumptions are needed for Scope-2 emissions about the GHG mix of electricity consumed by each city.

City-level GHG emissions are mostly self-reported using different inventory methods based on energy and fuel statistics and follow different protocols^5^. Among the different types of GHG emissions in cities, fossil fuel CO_2_ emissions are more robustly comparable between cities as they constitute the largest share of total GHG emissions and are estimated by more cities than non-CO_2_ gas emissions. Additionally, Scope-1 CO_2_ emissions from inventories could ultimately be compared to observation-based estimates using atmospheric CO_2_ measurements and tracer transport models^6,7^, provided emissions from power plants within a city territory are included in reported Scope-1 emissions.

Ancillary emission drivers data related to processes impacting the use of carbon fuels allow us to better understand differences in CO_2_ emission patterns between cities. Key drivers are population and Gross Domestic Product (GDP) per capita. For instance, analysis of the relationships between per-capita consumption and per-capita GDP was applied to energy consumption in cities by Creutzig *et al.*^1^, who derived a typology of urban energy use and mitigation potentials and highlighted city compactness and high gasoline prices as explanatory variables for lower energy use in affluent cities.

A comprehensive global city dataset with Scope-1 CO_2_ emissions with data related to emission drivers is still missing, although there are several regional datasets^8–13^ as well as recent efforts to estimate city carbon footprints using regional^14^ and national^15^ consumption-based models. Sorting out the best available information on data sources and a better understanding of the methods used to produce them help address inconsistencies across the set of cities studied; however, full traceability to the original data used in each city emission inventory is often not possible, and city-level CO_2_ emissions and driver-related data are not always consistent across space and time. Further, the scope of gases and sectors covered by the datasets are often uneven and activity data could have been scaled down from national data when local activity data are unavailable.

Here, we present a global dataset of Scope-1 city CO_2_ emissions based on CDP^16^, the carbon*n* Climate Registry of the Bonn Center for Local Climate Action and Reporting (http://carbonn.org), and a set of Chinese cities compiled by Beijing University (private communication). CO_2_ emission data are complemented with key ancillary data associated with emissions. The dataset includes 343 cities in nine geographic regions. The majority (88%) of these cities reported emissions between 2010–2015. When the original data reported total emissions from multiple GHGs, we developed a simple correction procedure to remove the contribution of methane (CH_4_). Scope-2 emission data are provided as additional information when available. Wherever possible, independent consistency checks of the data are performed. The final dataset of Scope-1 CO_2_ emissions and ancillary data is organized as a tab-separated values (TSV) file of dimensions 343 rows (cities) × 179 columns.

In the following, we describe the methodology used to produce the final dataset (Methods) and details about each individual dataset (Data Records). The Technical Validation section includes analyses to support the technical quality of the dataset. That is, the Quality Assurance/Quality Control (QA/QC) applied to data records of the same variable when different datasets include this variable, and the comparison of Scope-1 CO_2_ emissions from this study with independent datasets for US and Japanese cities.

## Methods

**Figure 1:**
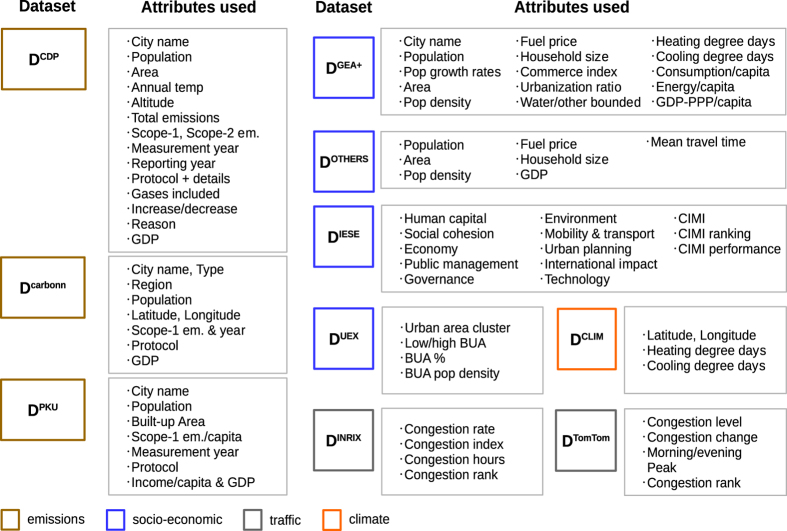
Source datasets and their attributes. Source datasets D* and their attributes used to construct the final dataset D^final^. Colors indicate type of dataset: brown (emissions), blue (socio-economic), gray (traffic-related), orange (climate-related).

**Figure 2:**
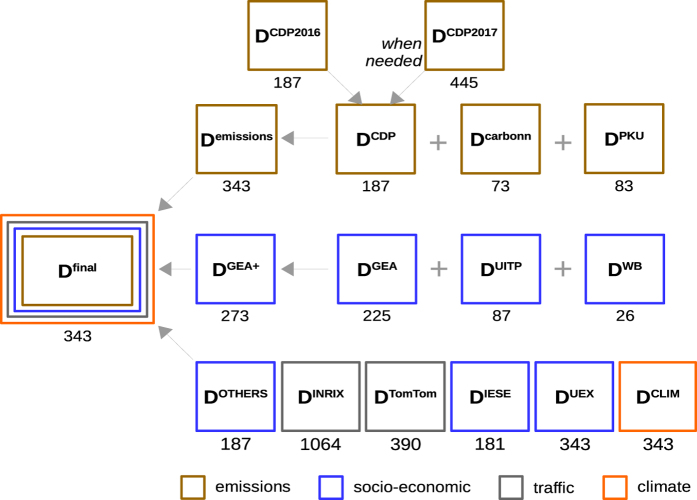
Flow chart of the steps in Methods to produce final dataset D^final^. Colors follow same scheme as in Fig. 1; numbers refer to the number of cities in each dataset D*.

Figure 1 shows the different datasets (D*) used to construct the final consolidated dataset of Scope-1 CO_2_ emissions and ancillary data. Table 1 lists each D* and the method steps in which they intervene. Figure 2 gives the flow chart followed to produce the final dataset from individual D*. Most data handling in the Methods (reading, processing, validating, visualizing, compiling, saving) was done in Jupyter notebooks (http://jupyter.org/, open-source web application for code, visualizations, etc.) using pandas DataFrames (http://pandas.pydata.org/), a data structure that allows data sorting, counting or conditional replacement to be executed on tabular data in lines of code. Table 2 (available online only) lists the attributes preserved in the final dataset, along with their source dataset and references.

### Step 1. Scope-1 GHG emissions from CDP

The 2016 edition of the CDP emissions dataset^16^ (D^CDP2016^) provides annual mean GHG emissions in CO_2_ equivalents from 187 cities in eight geographic regions. The CDP data are collected through an Online Response System that allows cities to report climate hazards, climate actions, targets and emission inventories. The emissions disclosed to CDP are provided directly by city governments. Cities first identify the inventory boundary and their emissions sources. Most cities use the scopes framework to report Scope-1 and Scope-2 emissions.

**Figure 3:**
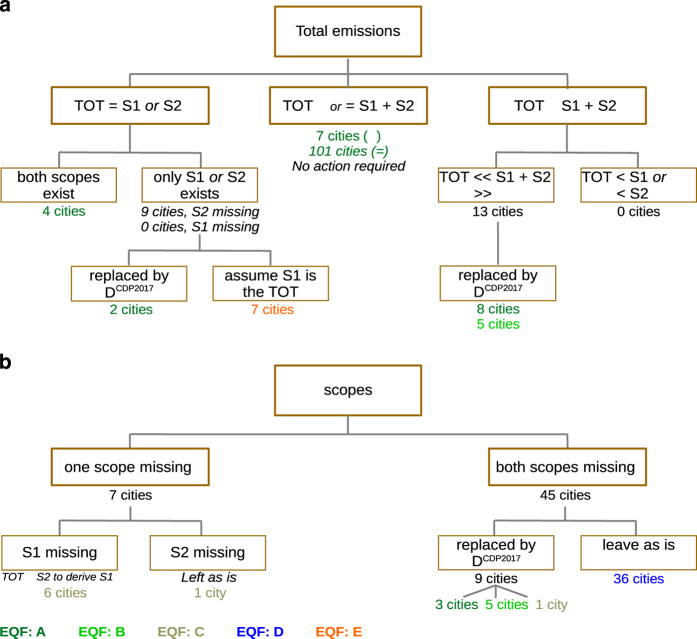
Assignment of Emissions Quality Flags in D^CDP^. Emissions quality flags (EQFs) assigned to D^CDP2016^ Scope-1 GHG emissions data in the case where both Scope-1 and Scope-2 emissions data are available. (**a**) EQFs are assigned based on the consistency between total emissions (TOT) and the sum of Scope-1 (S1) and Scope-2 emissions (S2). The ideal case with EQF = A is when TOT = S1+S2. Deviations from this expectation result in a hierarchy of EQFs according to the flowchart in the figure. (**b**) EQFs in the case where only one or no Scope-1 and Scope-2 GHG emissions data were available. A tolerance margin of ±15% was allowed for TOT ≈ S1 + S2. The number of cities for each check and the number of cities from D^CDP2017^ used as replacement data is indicated. Colors indicate the EQF assigned. Before replacing any data with D^CDP2017^, QA/QC checks were applied. Special case for Rotterdam: D^CDP2016^ data was EQF A (TOT = S1 + S2), but Scope-1 included power plant emissions, therefore the data were replaced by D^CDP2017^ values (EQF C, S2 missing).

Scope-1 are the direct territorial emissions from residential and industrial heating, transport, industrial sectors and power plants within the territory of cities^5^. Scope-2 are the emissions from purchased energy generated upstream from the city, mainly electricity^5^. Total Emissions are the sum of the two scopes. Emission data in D^CDP2016^ were examined manually to extract only Scope-1 emissions, after correcting for inconsistencies by either consulting with the city data providers or by comparing with a more recent version^17^ of the CDP data called D^CDP2017^. Based on quality control procedures comparing Scope-1, Scope-2 and total emissions (see Technical Validation), quality flags were assigned to the GHG Scope-1 emissions of each city. The quality-controlled data are denoted D^CDP^. Figure 3 shows the flowchart of the quality control of emissions in D^CDP^, summarized in Table 3 (detailed explanations in Technical Validation).

To calculate their emissions, cities collect data for a variety of sources, including city departments, their country’s national inventory, local utilities, statistics agencies, and universities. Methods such as scaling can be used for national data to estimate the cities inventory. Once cities have the appropriate data, they multiply the activity data by an emission factor associated with the activity being measured. Cities then compile all this information and provide CDP their inventory and a summary of their inventory data.

### Step 2. Separate CO_2_ emissions from non-CO_2_ GHGs

Since emissions of different greenhouse gases (GHGs) are included under Scope-1 emissions, we separated the fraction of emissions from fossil fuel and cement CO_2_ sources from other GHG contributions in D^CDP^. In total, 44 cities (25%) reported only emissions from fossil fuel and cement CO_2_, 6 cities did not specify the GHGs covered, 4 reported CO_2_ and CH_4_, and 133 included all GHGs, that is, CO_2_, CH_4_, N_2_O, HFCs, PFCs, SF_6_, and/or NF_3_ (see Table 4). We separated Scope-1 CO_2_ emissions from Scope-1 GHG emissions, f(G), with *G* = {CO_2_, CH_4_, N_2_O, HFC, PFC, SF_6_, NF_3_}, assuming *G* to be the set of CO_2_-equivalent GHGs.

$$G=\left\{{\mathrm{CO}}_{2},\;{\mathrm{CH}}_{4}-\text{eq},\;N_{2}O-\text{eq},\;\text{HFC}-\text{eq},\;\text{PFC}-\text{eq},\;{\mathrm{SF}}_{6}-\text{eq},\;{\mathrm{NF}}_{3}-\text{eq}\right\},$$

*α*_*i*_ be the GHG warming potential of gas $g_{i}\in G$ with *α*_*i*_ = 1 for *g*_*1*_ = CO_2_, and *f*(*G*) = *f*(*g*_*1*_,…, *g*_n_) the Scope-1 CO_2_-equivalent emissions (*f*) as a function of the set of GHGs considered. Then:

$$f\left(G\right)=f\left(g_{1}\right)+\alpha_{2}f\left(g_{2}\right)+\ldots +\alpha_{n}f\left(g_{n}\right)=f\left(g_{1}\right)-\sum_{i=2}^{n}\alpha_{i}f(g_{i})$$

Where *f(g*_*i*_) is the emission of each GHG, and *α*_*i*_ its warming potential. The emission of CO_2_ denoted by $f(g_{1})$ is expressed by:

$$f\left(g_{1}\right)=f\left(G\right)-\sum_{i=2}^{n}\alpha_{i}f(g_{i})$$

Neglecting emissions of other non-CO_2_ GHGs compared to those of CH_4_, denoted by $f\left(g_{1}\right)$:

(1)f(g1)≈f(G)−α2f(g2)

An upper bound of Scope-1 CO_2_ emissions were $f^{max}\left(g_{1}\right)$obtained by considering only CH_4_ emissions from waste in each city, approximated by national per capita methane waste emissions multiplied by city population, using country emissions data from the EDGAR inventory (Data Citation 1), country population from The World Factbook (2010 values, https://www.cia.gov/library/publications/the-world-factbook/rankorder/rankorderguide.html), and a GWP of 28 for CH_4_, time horizon 100 years^18^. We highlight that this is a simplification; however, deriving city-specific estimates from country-specific emission factors seems reasonable and agrees with the approach taken by UNFCCC for the official national reporting. Regarding whether per capita waste generation (solid waste and waste water) might differ between rural and urban areas, we assume that for OECD countries, there is only a minor difference since consumption and income levels are closely correlated. For emerging countries, the difference in per capita waste of rural and urban population could be larger or CH_4_ emissions from waste might not even be covered in all rural areas as managed, and this accounted for in the used CH_4_ emission dataset.

(2)fmax(g1)=f(G)−α2Fwaste(g2)ncountry⋅ncity

A lower bound of $f^{min}\left(g_{1}\right)$ Scope-1 CO_2_ emissions was obtained using the same approach, considering this time CH_4_ emissions from waste and from natural gas.

(3)fmin(g1)=f(G)−α2[Fwaste(g2)+Fgas(g2)]ncountry⋅ncity

Note that figures for national methane emissions from natural gas production should be scaled by the remaining fraction (*β*) to account for import and export. If the volume of domestic natural gas production, import and export are represented by *v*_*production*_, *v*_*import*_ and *v*_*export*_, respectively, then:

(4)β=(1−vexportvimport+vproduction)

And equation (3) becomes:

(5)fmin(g1)=f(G)−α2[Fwaste(g2)+Fgas(g2)⋅β]ncountry⋅ncity

Even after this correction, we likely overestimate urban CH_4_ emissions from the use of natural gas, since significant amounts of gas transported and consumed within the country is lost in extraction and mid-stream processes rather than only in the urban natural gas distribution grids and at the consumer level. The inferred CH_4_ emissions for each city, here denoted D^CH4^, were calculated and stored in an Excel file.

### Step 3. Integrate two other emission datasets focusing on developing countries

Most of the cities that disclosed their emissions to CDP are from USA, Canada, Australia, New Zealand (43%) and Europe (31%). Therefore, the D^CDP^ dataset lacks coverage for cities in developing regions, which limits the use of the data for global analysis and synthesis of differences across cities. To fill this gap, we collected two additional emission datasets: 1) Scope-1 GHG emissions reported by 48 large cities from the carbon*n* Climate Registry (D^carbonn^, http://carbonn.org) with 6 cities in Africa, 5 in East Asia, 10 in Latin America & Caribbean, 3 in North Africa, Middle East, West Asia, 19 in South Asia, and 18 in Southeast Asia, and 25 other cities for which we had ancillary data from Creutzig *et al.*^1^; 2) Scope-1 CO_2_ emissions from the new data compilation of 83 large cities in China from Peking University (D^PKU^). The emissions were grouped with D^CDP^ into a single dataset denoted D^emissions^ covering 343 cities, for which ancillary data was then gathered in the following steps. Note that D^CDP^, D^carbonn^ and D^PKU^ contained some ancillary data that we preserved (see Data Records section).

### Step 4. Match ancillary data with Scope-1 CO_2_ emissions

Ancillary data related to emissions were collected to match Scope-1 CO_2_ emissions. Spatial units of these ancillary data were checked for consistency with emissions data. The consistency of spatial and temporal units between ancillary data and emissions, and within ancillary data themselves, were checked and assigned a Quality Flag (see Technical Validation). Table 2 (available online only) gives the complete list of ancillary attributes and their different source datasets.

### Socio-economic and observational climate data

First, we used three datasets previously reported in Creutzig *et al.*^1^ (see Fig. 1): GEA^19^ (225 cities, henceforth D^GEA^), UITP (https://trid.trb.org/view/708144) (87 cities, henceforth D^UITP^), and WB^20^ data (26 cities, henceforth D^WB^). These datasets contain energy-use, other socio-economic and climate-related attributes. We integrated the GEA, UITP and WB datasets that comprise 273 cities into D^GEA+^, but this dataset only matched emissions from 57 cities from D^CDP^, 25 from D^carbonn^ and 35 from D^PKU^. In D^GEA+^, duplicate identical values of the same attribute in D^GEA^, D^UITP^ and D^WB^ were merged into one common column tagged with label ‘GEA+’. If the values were different, they were investigated further to resolve discrepancies (e.g. data entry errors) or left as is if the values were obtained from different sources; any such discrepancies are noted in the Data Records.

Altogether, the D^GEA+^ attributes include population, area, population density, national diesel and gasoline prices, average household size, bounding geographical features, a commerce index (https://www.scribd.com/document/17016734/MasterCard-Worldwide-Centers-of-Commerce-Index-2008), an urbanization index (http://www.un.org/en/development/desa/population/publications/pdf/urbanization/WUP2011_Report.pdf), and climate indices of heating and cooling degree days (HDD and CDD). HDD and CDD are commonly used to estimate the climate-dependent demand for energy needed to heat or cool a building, respectively, and are defined as the yearly sum of the difference between a base temperature *T*_*b*_ and daily temperature, *T*_*daily*_, whenever the daily temperature is lower (HDD) or greater (CDD) than *T*_*b*_:

(6)HDD=∑daysMAX(Tb−Tdaily,0)

(7)CDD=∑daysMAX(Tdaily−Tb,0)

In D^GEA^, D^UITP^ and D^WB^, HDD and CDD were given for a 5-year average over 2007–2011 for 13 base temperatures in increments of 0.5 °C (*T*_*b*_ = 12.5 °C–18.5 °C for HDD and *T*_*b*_ = 17 °C–23 °C for CDD). Those values were computed by the online software tool Degree Days.net (www.degreedays.net) created by BizEE Software (http://www.bizeesoftware.com/) using daily temperature data obtained from Weather Underground local weather stations in different cities (www.wunderground.com). The software returns monthly averages across the year range specified.

We merged D^GEA+^ with D^emissions^. Missing ancillary data in this merged dataset were then completed from external sources and merged again into D^emissions^ in the order listed below (for details on ancillary data types and methods, see Data Records).

### Externally sourced ancillary data to gap-fill GEA, UITP and WB

We obtained external ancillary data denoted as D^OTHERS^ to fill gaps in D^GEA+^ and to add new attributes related to emissions. Note that D^OTHERS^ data were not used to replace any data – the names of these independent, updated or new attributes were tagged with an ‘others’ label and added alongside existing attributes (themselves tagged with their dataset of origin). City population and area from multiple sources were obtained for all cities in D^emissions^. Diesel and gasoline prices provided in D^GEA+^ for year 2009^21^ were updated to 2014 values^22^ for all the cities in D^emissions^. Average household size was added for 119 out of 139 cities in D^emissions^ that did not have this attribute from D^GEA+^, and 48 D^GEA+^ values were updated. The mean travel time to work was obtained from external sources for 152 of the 187 CDP cities in D^emissions^. City area, population, average household size, and mean travel time were checked for spatial consistency before being included in D^OTHERS^. Gross Domestic Product (GDP) estimates (at Purchasing Power Parity (PPP) and nominal) along with population values for corresponding years, where available, were obtained manually from various external data sources (see Data Records). Altogether, GDP-PPP (nominal GDP) estimates were found for 153 (211) cities. The original GDP estimates given in D^PKU^ for all 83 Chinese cities were not checked against independent sources.

### New congestion-related data from INRIX and TomTom

Congestion-related data was obtained from INRIX^23^ (D^INRIX^) covering in total 1064 cities of which 118 matched D^emissions^, and from TomTom (D^tomTom^, https://www.tomtom.com/en_gb/trafficindex/), covering 390 cities of which 123 matched D^emissionss^.

### New socio-economic indicators from IESE

Socio-economic indicators related to transportation, environment, economy, technology, governance and human capital was obtained from the IESE Cities in Motion dataset^24^ based on 2015 data (D^IESE^) for 181 cities, of which 85 cities matched D^emissions^.

### Urban area expansion data

Urban area expansion in units of percentage of total built-up area (BUA) for low and high BUAs reflects the increase in the built environment due to activities related to urban life, such as housing construction. Here, we calculated 18 urban area expansion attributes pertaining to three different years (1990, 2000, 2014), denoted D^UEX^, from high-resolution satellite land cover imagery in 282 urban area clusters that correspond to 343 cities in D^emissions^. The D^UEX^ attributes relate to, for each year, high and low built-up areas (BUAs, km^2^), the BUA fraction (%) out of the total BUA for high and low BUAs, and low/high BUA population density per km^2^. D^UEX^ was derived from Landsat imagery collections, pre-processed by the Global Human Settlement (http://data.europa.eu/89h/jrc-ghsl-ghs_smod_pop_globe_r2016a) and built-up (http://data.europa.eu/89h/jrc-ghsl-ghs_built_ldsmt_globe_r2015b) grids, and population data from the Center for International Earth Science Information Network (CIESIN) Gridded Population of the World (GPWv4) (http://data.europa.eu/89h/jrc-ghsl-ghs_pop_gpw4_globe_r2015a). Please see the Data Records section for more details.

### CDD and HDD climate indices

Although D^GEA+^ had CDD and HDD indices for some cities, these indices were recomputed (D^clim^) from a global harmonized gridded climate dataset^25^ sampled for the grid-cell containing each city. D^clim^ contains HDD for a base temperature (*T*_*b*_) of 15.5 °C and CDD for a base temperature (*T*_*b*_) of 23 °C.

The dataset containing D^emissions^ merged with all ancillary datasets (D^GEA+^, D^INRIX^, D^TomTom^, D^IESE^, D^clim^, D^UEX^, and D^OTHERS^) is denoted D^final^ and has dimensions of 343 rows (cites) × 179 columns (first column after city name and definitions = Scope-1 GHG emissions, others = Scope-2 emissions, ancillary data, and quality flags). The data is publicly available on PANGAEA (Data Citation 2).

**Figure 4:**
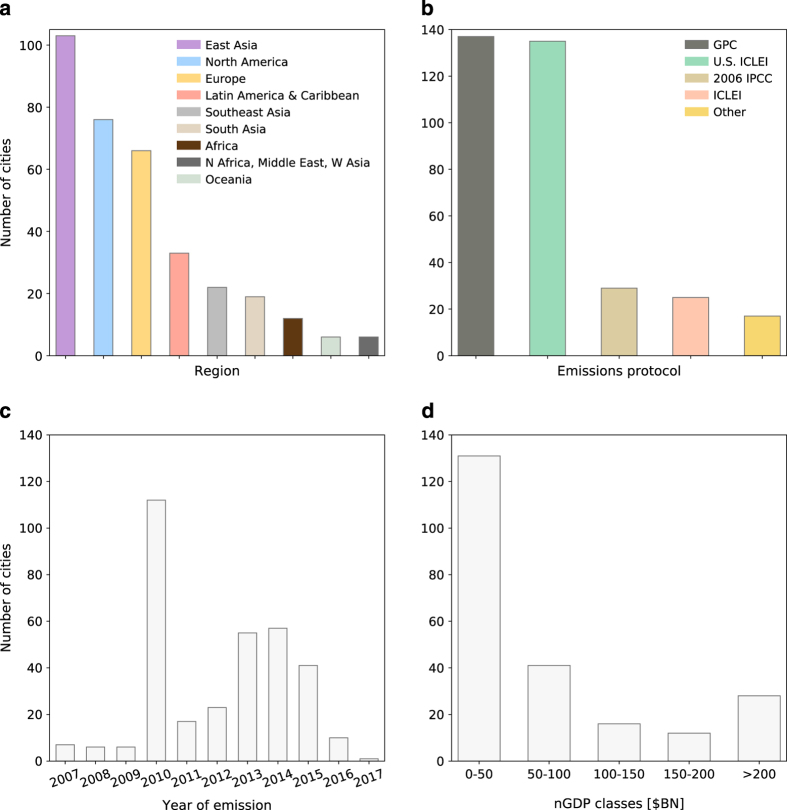
General characteristics of the final dataset. Barplots of the characteristics of the final dataset D^final^. (Top) Number of cities per geographic region (**a**) and per emissions protocol (**b**). (Bottom) Number of cities per emission year (**c**) and per nominal GDP (nGDP [$BN]) class where classes ranges are [0–50], (50–100], (100–150], (150–200], >200 (**d**).

**Figure 5:**
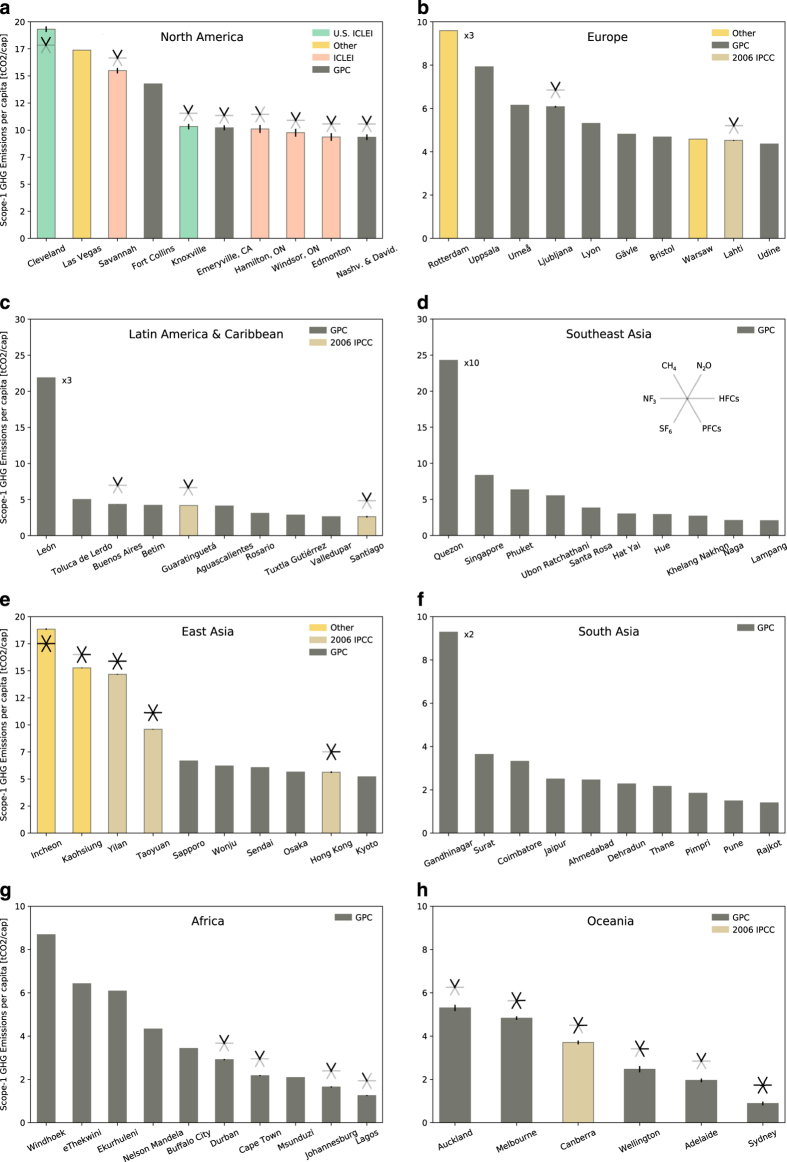
Scope-1 GHG emissions/capita for top-10 highest-emitting cities per capita. Barplots of the Scope-1 GHG emissions/capita in descending order in eight different geographical regions (**a–h**) colored by emissions protocol. Only the ten highest per capita emissions are shown for regions with more than ten cities. Four cities (Rotterdam, León, Quezon, Gandhinagar) with per capita emissions much larger than the other cities in their region were scaled down by a factor of 3, 3, 10, 2, respectively. Scale factor is denoted by a small annotation e.g. “×3” next to the bar.

To summarize the coverage of the different data contained in D^final^, Figure 4 shows the number of cities per geographic group, per primary emission calculation methodology/emission reporting protocol, per years of emissions estimate, and per nominal GDP class where classes ranges are [0–100], (100–200], (200–300], (300–400], > 400 $BN. Figures 5 shows Scope-1 GHG emissions per capita in eight geographic regions, sorted in descending order according to Scope-1/capita for the ten highest per capita emission values.

The new dataset D^final^ covers emissions and ancillary data in a wide range of countries and 83 cities in China, allowing data to be analyzed for differences between cities worldwide (e.g. low/high populations, different urban forms, different traffic and climate conditions, and different GDP). In the interest of “data discoverability”, we developed an interactive, web-based data visualization, available at the temporary URL https://katirg.github.io/GlobalCarbonCities/. Permanent visualization of D^final^ will be available in the Global Carbon Atlas at www.globalcarbonatlas.org in March, 2019.

### Code availability

The Jupyter notebooks (IPython version 5.1.0) used in this section are publicly archived with the final dataset on PANGAEA (Data Citation 2), along with the necessary input files (D^GEA+^, D^INRIX^, D^TomTom^, D^IESE^, D^clim^, D^UEX^, and D^OTHERS^). The notebooks import the following modules: pandas (version 0.22.0), numpy (version 1.14.0), matplotlib (version 2.1.2), and csv (version 1.0).

## Data Records

This section gives details of each data record in D^final^ as listed in Table 2 (available online only). Five data records related to Scope-1 emissions and three data records related to geo-descriptive attributes were set as common columns in D^final^ to avoid redundancy. The five common Scope-1-related columns are:

### ‘Scope-1 GHG emissions [tCO2 or tCO2-eq]’ [float]

Common column to store Scope-1 GHG emissions values in D^final^. See ‘Scope-1 GHG emissions units’ for an explanation of the units used. *CDP*: Scope-1 GHG values exist for 151 out of 187 cities. According to the Emissions Quality Flag (EQF) (see Technical Validation), good quality Scope-1 GHG emissions (EQF A, B or C) were found for 144 of these cities. *Carbonn*: Scope-1 GHG values were obtained for 73 cities. *PKU*: Scope-1 GHG values were obtained for 83 cities.

### ‘Scope-1 source dataset’ [string]

Common column to store the name of the dataset corresponding to the Scope-1 emissions in D^final^, i.e., ‘CDP’, ‘carbonn’, or ‘PKU’.

### ‘Scope-1 GHG emissions units’ [string]

Common column to store the units of the Scope-1 GHG emissions values in D^final^. *CDP*: Units ‘tCO_2_’ are used for cities that reported only CO_2_ (refer to the ‘Gases included (CDP)’ attribute), and units ‘tCO_2_-eq’ are used for cities that specified gases other than CO_2_ or did not specify whether non-CO_2_ gases were included. *Carbonn*: units ‘tCO_2_-eq’ are used since a gas species breakdown was not available. *PKU*: units ‘tCO_2_’ are used for all cities since emissions related to CO_2_ only.

### ‘Year of emission’ [string]

Common column to store the year of the Scope-1 GHG emissions values in D^final^. *CDP*: emission years ranged from 1990–2016, with 88% of the data reported for the period 2010–2015. *Carbonn*: emission years ranged from 1994–2017, with 78% of the data reported for the period 2010–2015. *PKU*: all emissions were estimated for the year 2010.

### ‘Emissions protocol’ [string]

Common column to store the emissions protocol used to obtain the Scope-1 GHG emissions values in D^final^.

*CDP*: The protocols used to estimate emissions in D^CDP^ are described in the CDP submission guidelines (https://b8f65cb373b1b7b15feb-c70d8ead6ced550b4d987d7c03fcdd1d.ssl.cf3.rackcdn.com/cms/guidance_docs/pdfs/000/000/507/original/CDP-Cities-Guidance-2017.pdf?1484751625; current as of Jan 18, 2017). The majority of cities report their inventory to CDP using the Global Protocol for Community-Scale Greenhouse Gas Emission Inventories (GPC)^5^ launched in 2014 by C40, WRI and ICLEI, after a pilot in 2011 with 35 cities. The GPC aims to enable a consistent, transparent and internationally recognized approach for cities to measure and report emissions, allowing for credible comparison and aggregation across timescales and geographies. Cities that commit to the Compact of Mayors, now the Global Covenant of Mayors for Climate and Energy, must complete an emissions inventory using the GPC methodology.

Within GPC, cities can choose to either report BASIC (Scope 1 + Scope 2 + some waste Scope 3 emissions) or BASIC+ emissions (includes all Scope-1, Scope 2 and Scope 3 emissions). The breakdown was as follows:

1. Global Protocol for Community-Scale Greenhouse Gas Emissions Inventories (GPC), (WRI, C40 and ICLEI), 64 cities (34%). Comprises emissions from stationary energy, transportation, waste, Industrial Processes and Product Use (IPPU), Agriculture, Forestry and Other Land Use (AFOLU), Total Scope 1 (Territorial emissions), and Total BASIC emissions.

2. International Emissions Analysis Protocol (ICLEI), 18 cities (10%). Comprises emissions from stationary energy, transport, fugitive emissions, industrial processes, agriculture, land use, land use change and forestry, solid waste disposal, wastewater treatment and discharge, and other sources.

3. 2006 IPCC Guidelines for National Greenhouse Gas Inventories, 29 cities (16%). Comprises emissions from energy, IPPU, AFOLU, waste and other sectors.

4. U.S. Community Protocol for Accounting and Reporting of Greenhouse Gas Emissions (ICLEI), 26 cities (15%). Comprises emissions from built environment, transportation and other mobile sources, solid waste; wastewater and water, agricultural livestock, and upstream impacts of community-wide activities.

5. “Other”, 50 cities (27%). This category includes combinations or subsets of methodologies, or propitiatory methodologies specific to a region/city. Comprises emissions from buildings, water, waste, transport/residential/commercial/industrial/ institutional use, stationary combustion, mobile combustion, industrial processes, waste, and any other classification.

*Carbonn*: cities reported emissions based on the GPC protocol (http://e-lib.iclei.org/wp-content/uploads/2015/12/cCR2015_5Year_Report.pdf).

*PKU*: Scope-1 CO_2_ emissions were calculated from direct energy consumption statistics^26^ with CO_2_ emission factors of diesel oil, coal gas, natural gas, and liquefied petroleum gas calculated according to the guidelines of IPCC 2006^27^.

The three common geo-descriptive columns are:

### ‘City name’ [string]

Consistent city name assigned to each city in D^final^. Ambiguous city names were corrected (e.g. ‘Peterborough’ in D^CDP^ was renamed to ‘Peterborough, ON’ to disambiguate from ‘Peterborough’ in the UK in D^GEA^).

### ‘Country’ [string]

Consistent country name used in D^final^.

### ‘Region’ [string]

A geographic region assigned to each city in D^final^ using the regions defined in D^carbonn^.

### 1. D^CDP^ dataset

Scope-1 CO_2_ emissions separated as described in Methods from total GHG emissions reported by cities in D^CDP^ were estimated between 1990–2016, with 88% of the data being in the period 2010–2015. Some cities also reported change in their emissions between the most recent and previous reporting periods, explanations for this change, methodology details, and gases included, as well as ancillary data such as population, boundary definition, land area, mean annual temperature, mean altitude, and Gross Domestic Product (GDP). The following records were obtained from D^CDP^.

### ‘City name (CDP)’ [string]

Name provided by each city.

### ‘Definition (CDP)’ [string]

Boundary of the city provided by each city. Boundary types are diverse and correspond to metropolitan area for 25 cities, administrative boundary of a local government: 147; combination of administrative divisions: 2; geopolitical Boundary - physical areas over which local government has jurisdictional control: 3; municipal boundary: 1; Other boundary types: 4 cities.

### ‘Reporting year (CDP)’ [integer]

The year when a city reported data to CDP, that is, 2016 for 162 cities and 2017 for 25 cities.

### ‘City location (CDP) [degrees]’ [float]

Latitude and longitude coordinates provided by each city. The following discrepancies were noted in these coordinates. Wellington referred to Wellington, Florida instead of Wellington, New Zealand. Moita in Portugal did not localize to the city. We set the Moita city near Lisbon, since the population (66,029) and emissions (96,508 tCO2-eq) in D^CDP^ match the recent report from (Ricardo Energy & Environment, 2017). Also, according to the Instituto Nacional de Estatística Portugal, the population of Moita near Lisbon is 66,029 (https://en.wikipedia.org/wiki/Moita).

### ‘Population (CDP)’ [integer]

Reported city population.

### ‘Population year (CDP)’ [integer]

Year corresponding to reported city population data.

### ‘City area (CDP) [km2]’ [float]

City land area available for 184 out of 187 cities in D^CDP^.

### ‘Gases included (CDP)’ [string]

Gases included in the reported emissions. Out of 187 cities, 44 included only CO_2_, 6 cities did not specify, 4 cities included CO_2_ and CH_4_, and the remaining 133 cities included CO_2_, CH_4_ plus a combination of one or more of N_2_O, HFCs, PFCs, SF_6_, and/or NF_3_ (see Table 4).

### ‘Methodology details (CDP)’ [string]

Some cities provided comments relating to how they estimated their emissions within the specified protocol.

### ‘Increase/Decrease from last year (CDP)’ [string]

Change in emissions based on previous reporting year. The majority (88 [47%]) of cities reduced their emissions with respect to the previous reporting year. Emissions increased for 36 (19%) cities, and stayed the same for 12 (6%) cities. Of the remaining cities, 24 (13%) cities were reporting their emissions for the first time in 2016 (thus had no previous comparison year), 7 (4%) cities reported “other” to indicate that their emission data was not directly comparable to previous estimates due to protocol differences, and 20 cities (11%) did not specify.

### ‘Reason for increase/decrease in emissions (CDP)’ [string]

The interested reader may refer to the original D^CDP2016^ dataset to explore the reasons provided for changes in emissions. Most reasons tended to be due to a methodological change, population growth, change in policy, or increase in renewable energy and electricity.

### ‘Scope-2 (CDP) [tCO2-eq] ‘ [float]

Scope-2 GHG emissions from D^CDP^ in units of metric ton CO_2_-eq. Note that some cities reported only CO_2_, effectively making this unit tCO_2_ (refer to the ‘Gases included (CDP)’ attribute). Scope-2 emissions exist for 141 out of 187 cities which were of good quality (i.e. Emissions Quality Flag A, B or C).

### ‘Total Emissions (CDP) [tCO2-eq]’ [float]

The city total CO_2_ emissions reported in 2016 in units of metric ton CO_2_-eq. Note that some cities reported only CO_2_, effectively making this unit tCO_2_ (refer to the ‘Gases included (CDP)’ attribute). Depending on the methodology selected, Scope-1 and Scope-2 emissions are included in total emissions.

### ‘Average altitude [m]’ [integer]

Average altitude of the city in meters above sea level, available for 168 out of the 187 cities in D^CDP^. Values provided by source for D^CDP^ cities.

### ‘Average annual temperature [degrees Celsius] (CDP)’ [float]

Average annual temperature in degrees C, available for 177 out of the 187 cities in D^CDP^.

### ‘GDP (CDP) [multiple units]’ [integer]

GDP of the city in the currency reported by the city (given in next column).

### ‘GDP unit (CDP)’ [string]

Currency of the GDP value.

### ‘GDP year (CDP)’ [integer]

The year for which the GDP value was obtained.

### ‘GDP source’ [integer]

Source of reported GDP data.

### ‘CDP2016 data edited (CDP)’ [boolean]

For cities whose CDP2016 data were replaced by CDP2017 values; reasons are indicated in this column. Please see the Technical Validation section.

### ‘Emissions Quality Flag (CDP)’ [string]

Quality flags (A–E) assigned to emissions as described in section ‘Technical Validation’.

### ‘S1 lower bound (CDP) [tCO2]’ & ‘S1 upper bound (CDP) [tCO2]’ [float]

Lower and upper bound estimates of Scope-1 emissions resulting from the methane corrections described in Methods Step 2.

### ‘S1 mean (CDP) [tCO2]’ [float]

Lower and upper bound estimates of Scope-1 emissions resulting from the methane corrections described in Methods Step 2.

### ‘TOT lower bound (CDP) [tCO2]’ & ‘TOT upper bound (CDP) [tCO2]’ [float]

Lower and upper bound estimates of total emissions resulting from the methane corrections described in Methods Step 2.

### ‘TOT mean (CDP) [tCO2]’ [float]

Lower and upper bound estimates of Total emissions resulting from the methane corrections described in Methods Step 2.

### ‘Scope fraction (CDP)’ [float]

Defined as (Scope 1 + Scope 2)/Total emissions. See ‘Quality Flags’ section and Figure 3.

### 2. D^carbonn^ dataset

Scope-1 GHG emissions from 73 cities selected in D^carbonn^ (see Methods) were estimated for years in the period 1994–2017, with 78% of the data in 2010–2015. Emissions correspond to all activities occurring throughout the local government’s entire geographic area (http://e-lib.iclei.org/wp-content/uploads/2015/12/cCR2015_5Year_Report.pdf). Cities also reported ancillary data such as city type, population, GDP and nominal GDP. The following records were obtained from D^carbonn^.

### ‘City name (carbonn)’ [string]

City name.

### ‘Definition (carbonn)’ [string]

City type: Municipality: 58; Independent municipality: 7; Special municipality/Federal district: 3; State/Region: 2; Independent intercommunality: 1; Intercommunality:1; Sovereign city-state: 1.

### ‘Population (carbonn)’ [integer]

City population.

### ‘Population year (carbonn)’ [integer]

Year corresponding to reported city population.

### ‘GDP (carbonn) [multiple units]’ [float]

GDP of the city in the units reported by the city (see next column).

### ‘GDP unit (carbonn)’ [string]

Currency unit of GDP.

### ‘GDP year (carbonn)’ [integer]

Year corresponding to reported GDP.

### ‘nGDP (carbonn) [multiple units]’ [float]

Nominal GDP in units specified in ‘GDP unit (carbonn)’ above.

### ‘Average altitude [m]’ [integer]

Average altitude of the city, in meters above sea level, available for 2 out of the 73 cities in D^carbonn^.

### 3. D^PKU^ dataset

All of the emissions reported by the 83 cities in D^PKU^ correspond to the year 2010. Cities also reported ancillary data such as urban population, built-up area, GDP and income per capita. The following records were obtained from D^PKU^.

### ‘City name (PKU)’ [string]

City name.

### ‘Urban population (PKU)’ [integer]

Urban population.

### ‘Built-up area (PKU) [km2]’ [float]

Built-up area was defined by interpreting Landsat images for each city in D^PKU^ (with spatial resolution of 30 meters).

### ‘GDP (PKU) [10000 RMB]’ [integer]

GDP of the city in renminbi currency units.

### ‘Income per capita (PKU) [RMB]’ [float]

Per capita income of the city in renminbi currency units.

### ‘CO2 emissions per capita (PKU) [tCO2/capita]’ [float]

Residential CO_2_ emissions provided by source.

### 4. D^GEA+^ dataset

As explained in Methods, D^GEA+^ is the fusion of D^GEA^, D^UITP^ and D^WB^ containing the following attributes:

### ‘City name (GEA)’ [string]

City name as given in D^GEA^.

### ‘City name (UITP)’ [string]

City name as given in D^UITP^.

### ‘City name (WB)’ [string]

City name as given in D^WB^.

### ‘Definition (WB)’ [string]

City type for D^WB^ cities: City: 12; Greater metropolitan area: 3; Government Administered Area (Province): 3; City and County: 1; County: 1.

### ‘Study year (WB)’ [integer]

Study year for emissions for D^WB^ cities (1998–2006).

### ‘Population (GEA)’ [float]

City population from D^GEA^, available for 41 of the 187 cities in D^CDP^.

### ‘Population (UITP)’ [integer]

City population from D^UITP^, available for 42 of the 187 cities in D^CDP^.

### ‘Population (WB)’ [integer]

City population from D^WB^, available for 15 of the 187 cities in D^CDP^.

### ‘Population year (WB)’ [integer]

Year corresponding to city population in D^WB^, available for 15 of the 187 cities in D^CDP^.

### ‘Population *year* (WB)’ [integer]

Population of urban agglomerations in *year* = 1950, 1990 and 2010 from D^WB^, available for 15 of the 187 cities in D^CDP^.

### ‘Population growth rate *year1-year2* (WB) [people/60years and people/20 years]’ [float]

Population growth rate between *year1* = 1950 and 1990 and *year2* = 2010 from D^WB^, available for 15 of the 187 cities in D^CDP^.

### ‘City area (GEA) [km2]’ [float]

City area from D^GEA^, available for 30 of the 187 cities in D^CDP^.

### ‘City area (WB) [km2]’ [float]

City area from D^WB^, available for 15 of the 187 cities in D^CDP^. Used Wikipedia values corresponding to definition of the city.

### ‘Population density (GEA) [people/km2]’ [float]

Population per area from D^GEA^, available for 41 of the 187 cities in D^CDP^.

### ‘Population/sqrt(area) (GEA) [people/km]’ [float]

Population per sqrt(area) from D^GEA^, available for 30 of the 187 cities in D^CDP^.

### ‘Population density (UITP) [people/km2]’ [float]

Population per area from D^UITP^, available for 42 of the 187 cities in D^CDP^.

### ‘Population density (WB) [people/km2]’ [float]

Population per area from D^WB^, available for 15 of the 187 cities in D^CDP^.

### ‘Population/sqrt(area) (WB) [people/km]’ [float]

Population per sqrt(area) from D^WB^, available for 15 of the 187 cities in D^CDP^.

### ‘Weather station ID (GEA+)’ [string]

The ID of the weather station used to calculate HDD and CDD, available for 56 of the 187 cities in D^CDP^. One city in D^GEA+^ (Brasília) was missing a weather station ID.

### ‘HDD 15.5 C (GEA+) [degrees C×days]’ [integer]

HDD values for a base temperature of 15.5 °C obtained from the weather station specified in the ‘weather_station_id’ attribute of D^GEA+^. HDD values across D^GEA^, D^UITP^ and D^WB^ were the same for all cities except Sao Paulo in D^UITP^, which disagreed with the values in the other two datasets. The D^UITP^ values were considered anomalous and were removed. No HDD data were available for one city (Brasília) in D^GEA+^.

### ‘CDD 23 C (GEA+) [degrees C×days]’ [integer]

CDD values for a base temperature of 23 °C obtained from the weather station specified in the ‘weather_station_id’ attribute of D^GEA+^. CDD values across D^GEA^, D^UITP^ and D^WB^ were the same for all cities except Sao Paulo in D^UITP^, which disagreed with the values in the other two datasets. The D^UITP^ values were considered anomalous and were removed. No CDD data were available for one city (Brasília) in D^GEA+^.

These base temperature values for HDD and CDD were selected since it was shown in a 2015 study^1^ that these temperature thresholds were the most predictive of energy use, given the data available. (Energy use increases linearly with decreasing temperature below 20 °C, and increases strongly nonlinearly with increasing temperatures above 30 °C^28^).

### ‘Total final consumption per capita (GEA) [GJ/capita/yr]’ [float]

Total final energy consumption in GJ/capita/yr. Provided by source.

### ‘Energy per capita CO2 (WB) [tCO2-eq /capita/yr]’ [float]

Per capita emissions for energy use in tCO_2_-eq/capita/yr, excluding aviation and marine sources. Provided by source.

### ‘Diesel price (GEA+) [USD/liter]’ [float]

November 2008 diesel price (national values)^21^. In the final consolidated dataset, these values were not used; updated 2014 values^22^ were used instead (see Methods Step 4).

### ‘Gasoline price (GEA+) [USD/liter]’ [float]

November 2008 gasoline price (national values)^21^. In the final consolidated dataset, these values were not used; updated 2014 values^22^ were used instead (see Methods Step 4).

Fuel prices for two cities (Rio de Janeiro and Sao Paulo) in D^UITP^ were anomalous compared with the corresponding D^GEA^ and D^WB^ values; cross-checking with the reference cited in the datasets revealed that these D^UITP^ values were incorrect and they were therefore removed.

### ‘Household size (GEA+) [people/household]’ [float]

Average number of persons per household, obtained from multiple sources, available for 48 of the 187 cities in D^CDP^. The following discrepancies/uncertainties were noted for household size in D^GEA+^:

*Sydney*: The 2011 household size reported in D^GEA+^ corresponds to the population of the Greater Sydney Area, whereas the corresponding city population in D^emissions^ corresponds to the ‘City of Sydney’.

*Stockholm*: It could not be confirmed that household size in D^GEA+^ represents the corresponding city population in D^emissions^. Household size in D^OTHERS^ was estimated by a manual calculation based on values from Statistics Sweden (http://www.statistikdatabasen.scb.se/pxweb/en/ssd/START__BE__BE0101__BE0101S/HushallT03/?rxid=5b1086fd-d703-4c03-8791-3c76c740804f).

*Lisbon*: It appears that the 2011 population for household size in D^GEA+^ does not match the corresponding city population in D^emissions^.

*Melbourne*: The household size reference link in D^GEA+^ is actually for Sydney.

*Seoul*. The household size reference link in D^GEA+^ is invalid.

It was also found that the household size source reference no longer exists for 14 of the 57 cities in D^GEA+^ that matched cities in D^CDP^.

### ‘Household size year (GEA+)’ [integer]

Year corresponding to household size values.

### ‘Household size source (GEA+)’ [string]

Reference corresponding to household size values obtained from multiple sources.

### ‘Household size comment (GEA+)’ [string]

Comments regarding household size computations.

### ‘Urbanization ratio (GEA+) [percent]’ [float]

Percentage of Population Residing in Urban Areas in 2005 (http://www.un.org/en/development/desa/population/publications/pdf/urbanization/WUP2011_Report.pdf), available for 57 of the 187 cities in D^CDP^.

### ‘Center of commerce index (GEA+) [dimensionless]’ [float]

Center of commerce index (https://www.scribd.com/document/17016734/MasterCard-Worldwide-Centers-of-Commerce-Index-2008), obtained by considering 43 indicators and 74 sub-indicators across seven dimensions (e.g. economic and business-related, legal and political framework, city livability), available for 33 of the 187 cities in D^CDP^.

### ‘Water bounded (GEA+)’ [boolean]

Indicates if city is bounded by water (0=no, 1=yes). Source: derived visually from Google maps, available for 51 of the 187 cities in D^CDP^.

### ‘Other bounded (GEA+)’ [string]

Indicates type of bounding geography (‘Big Lake, Mountains’, ‘Major River’, ‘Major River Junction’, ‘Marshland?’, ‘Mountains’, ‘Near coast, non on – small river, ‘River’). Despite the title of this column, strings for the geographical features are used as values, not 0 or 1. Source: derived visually from Google maps, available for 18 of the 187 cities in D^CDP^.

### ‘GDP-PPP/capita (GEA) [USD/year]’ [float]

GDP per capita per year at Purchasing Paring Parity (PPP) provided by D^GEA^.

### ‘GDP-PPP/capita (UITP) [USD/year]’ [float]

GDP per capita per year (PPP) provided by D^UITP^.

### ‘GDP-PPP/capita (WB) [USD/year]' [float]

GDP per capita per year (PPP) provided by D^WB^.

### ‘Ancillary from GEA+‘ [boolean]

Computed for each city in D^emissions^ to indicate whether the city has corresponding ancillary data in D^GEA+^ (value = 1) or if no matching city exists in D^GEA+^ (value NaN).

### 5. D^INRIX^ data records

The following congestion-based attributes were obtained from D^INRIX^. The attributes relate to congestion rate, defined as the ratio of the total drive time in congested vs. free flow traffic, in percent.

### ‘Peak hours spent in congestion (INRIX) [hours]’ [integer]

The average peak period congestion rate is applied to travel times to derive daily time spent in peak period congestion. The average number of hours spent in congestion during peak hours is then estimated assuming 240 working days a year.

### ‘Average congestion rate (INRIX) [percent]’ [float]

Average of seven congestion rates: peak periods on highways in and out of the city; peak periods within a city; day time travel on highways in and out of a city; day time travel within a city; late night on highways in and out of a city; late night within a city; weekend travel on all roads.

### ‘Congestion rank (INRIX) [dimensionless]’ [integer]

Determined by the number of peak hours that drivers spent in congestion in 2016.

### ‘INRIX congestion index (INRIX) [dimensionless]’ [float]

The seven congestion rates (see above) weighted by relative volumes to reflect typical driving patterns, which is then weighted by the Median Travel Time, effectively adjusting the congestion rate by the city’s size and associated average journey times.

### 6. D^TomTom^ dataset

The following congestion-based attributes were obtained from D^TomTom^. The attributes relate to congestion level, defined as the percent increase in overall travel times when compared to uncongested traffic.

### ‘Congestion level (TomTom) [×100 percent]’ [integer]

Increase in overall travel times when compared to uncongested traffic. Travel times were calculated using speed measurements on individual road segments and entire networks in TomTom’s historical traffic database, weighted by the number of measurements.

### ‘Congestion change (TomTom) [×100 percent]’ [integer]

Percent change in congestion level compared to the previous year.

### ‘Morning peak (TomTom) [percent]’ [integer]

Percent increase in travel time compared with uncongested traffic during morning peak times (defined per city based on real traffic measurements).

### ‘Evening peak (TomTom) [percent]’ [integer]

Percent increase in travel time compared with uncongested traffic during evening peak times (defined per city based on real traffic measurements).

### ‘Congestion rank (TomTom) [dimensionless]’ [integer]

Ranking based on Congestion Level; applied only to cities with population greater than 800,000.

### 7. D^IESE^ dataset

Thirteen socio-economic indicators were obtained from D^IESE^: Economy, Environment, Governance, Human capital, International impact, Mobility and transportation, Public management, Social cohesion, Technology, Urban planning, the Cities in Motion index (CIMI), CIMI ranking, and CIMI performance. These indicators reflect the sustainability and standard of living of a city.

The IESE indicators include both city and national level variables from several sources with objective measures (e.g., GPD per capita) as well as subjective measure (e.g., perception about traffic). The calculation of the synthetic indicators produces values that were standardized in order to note the differences amongst the various cities between positions more intuitively. In order to modify the scale of each city, the city with the highest score (first in the ranking) was assigned the number 100. Cities with a high performance (H) are considered to be those with an index greater than 90; relatively high (RH), between 60 and 90; average (A), between 45 and 60; and low (L), below 45.

### ‘Economy (IESE) [dimensionless]’ [integer]

The IESE economy index includes aspects that promote economic development. This index takes into account four positively-contributing indicators – labour productivity calculated as GDP/working population [thousands], number of headquarters of publicly traded companies, percentage of 18 to 64-year-old population who are new entrepreneurs or owners/managers of a new business [per capita], and gross domestic product [millions USD at 2014 prices], and two negatively-contributing indicators – number of calendar days needed so a business can operate legally, and ease of starting a business [rank].

### ‘Environment (IESE) [dimensionless]’ [integer]

The IESE environment index aims to reflect environmental sustainability/sustainable development. This index takes into account two positively-contributing indicators – percentage of the population with access to the water supply, and environmental performance index (1 = poor to 100 = good), and six negatively-contributing indicators – CO_2_ emissions (fossil fuel burning and cement manufacture [kt]), CO_2_ emission index, methane emissions arising from human activities (e.g. agriculture, industrial methane production [kt CO_2_-eq]), PM2.5 (amount of particles [annual mean] in the air whose diameter is less than 2.5 μm), PM10 (amount of particles [annual mean] in the air whose diameter is less than 10 μm), and pollution index.

### ‘Governance (IESE) [dimensionless]’ [integer]

The IESE governance index aims to reflect the effectiveness, quality and sound guidance of state intervention. This index takes into account five positively-contributing indicators: the strength of legal rights for borrowers and lenders, corruption perceptions index (0 = very corrupt to 100 = very transparent), number of functions of the city’s innovation department, range of government Web services, and whether a city has an open data platform.

### ‘Human capital (IESE) [dimensionless]’ [integer]

The IESE human capital index aims to reflect a city’s talent and ability to create/support/retain it. The index takes into account seven indicators, considered as positive contributors to the index: the proportion of the population with secondary education, the number of business schools, the international movement of higher-level students, the number of universities, the number of museums per city, the number of art galleries per city, and the expenditure on leisure and recreation [millions USD at 2014 prices].

### ‘International impact (IESE) [dimensionless]’ [integer]

The IESE international impact index aims to reflect the international renown of a city (effectiveness of a city’s brand, foreign investment attractiveness, etc.). This index takes into account four positively-contributing indicators: the number of international tourists, the number of passengers who travel with airlines, number of hotels per capita, and the number of international conferences and meetings hosted in a city, and one negatively-contributing indicator – ranking of cities according to the number of photos taken in the city and uploaded to Panoramio (community for sharing photographs online; low value = most photographed).

### ‘Mobility and transportation (IESE) [dimensionless]’ [integer]

The IESE mobility and transportation index aims to reflect how well a city facilitates movement and access to public transportation services. This index takes into account three positively-contributing indicators – the number of metro stations per city, number of arrival and departure flights in a city, means of transportation (increases with increasing public transportation options), and four negatively-contributing indicators – traffic index (time spent in traffic and the dissatisfaction this generates, plus estimates of CO_2_ consumption and other traffic system inefficiencies), traffic inefficiency index (high values represent high rates of inefficiency in driving, such as long journey times), number of road accidents per 100,000 inhabitants, and commute index (considering travel time to work).

### ‘Public management (IESE) [dimensionless]’ [integer]

The IESE public management index aims to reflect the administration’s efficiency. This index takes into account five positively-contributing indicators – total reserves [millions USD], reserves per capita [millions USD], number of embassies per city, Twitter users listed in prominent Twitter directories [unit of thousands], and two negatively-contributing indicators – total tax rate paid by businesses, and sales tax.

### ‘Social cohesion (IESE) [dimensionless]’ [integer]

The IESE social cohesion index aims to reflect the degree of social interaction and sense of belonging to a common situation or project. This index takes into account five positively-contributing indicators – the ratio of death per 100,000 inhabitants, crime rate, health index, unemployment rate (number of unemployed / labor force), the Gini index (calculated from the Gini coefficient (https://en.wikipedia.org/wiki/Gini_coefficient), varies from 0 to 100, with 0 being a situation of perfectly equitable income distribution and 100 that of perfect inequality), price of property as percentage of income) – and one negatively-contributing indicator, the ratio of women workers in the public administration.

### ‘Technology (IESE) [dimensionless]’ [integer]

The IESE technology index aims to reflect the quality of life achieved in society or the potential quality of life. This index takes into account nine positively-contributing indicators: the number of broadband subscribers, number of broadband users, number of IP addresses per capita, number of Facebook users per capita, number of mobile phones per capita, the quality of the city council’s website (the commitment of its information technology policy, support for the development of local businesses and other technology initiatives, 0 = poor, 5 = best), innovation index (0 = poor, 60 = best), number of smartphones per capita, and the number of wireless access points.

### ‘Urban planning (IESE) [dimensionless]’ [integer]

The IESE urban planning index aims to reflect the habitability of a city in terms of e.g. quality of health infrastructure, housing policies, design of public spaces. This index takes into account four positively-contributing indicators – percentage of the population with access to sanitation facilities, number of bicycle shops per capita, number of architecture firms per capita, and the number of cycling enthusiasts per capita, and one negatively-contributing indicator – number of people per household.

### ‘CIMI (IESE) [dimensionless]’ [integer]

The IESE Cities in Motion Index (CIMI) is a weighted aggregation of the 10 indices described above: economy: 1; human capital: 0.4814; international outreach: 0.6212; urban planning: 0.841; environment: 0.6215; technology: 0.3763; governance: 0.4047; social cohesion: 0.5941; mobility and transportation: 0.4707; and public management: 0.571.

### ‘CIMI ranking (IESE) [dimensionless]’ [integer]

The IESE CIMI ranking is a 1–181 ranking (since there are 181 cities) of the CIMI, where 1 = highest, 181 = lowest.

### ‘CIMI performance (IESE) [dimensionless]’ [string]

The IESE CIMI performance classifies a city’s performance based on its CIMI: high (H, CIMI > 90); relatively high (RH, CIMI between 60–90); average (A, CIMI between 45–60); low (L, CIMI < 40).

### 8. D^clim^ Heating Degree Days and Cold Degree Days

The cities in D^emissions^ that are not part of D^GEA+^ do not have climate indices, therefore we calculated average HDD and CDD over the same period (2007–2011) as in D^GEA+^ for all 343 cities in D^emissions^ for the optimal temperature thresholds of 15.5 °C for HDD and 23 °C for CDD, as explained above. The temperature data were based on the three-hourly ERA-interim reanalysis available from 1979 and updated in real time^29^, sampled at a spatial resolution of ≈ 80 km. Calculating climate indices from gridded data that is treated homogeneously allows comparison of different locations without possible biases such as weather station locations within a city. However, at the relatively coarse resolution of the grid, coastal and orographic effects on surface temperature may have been missed^25^. The city locations were obtained from the latitude and longitude coordinates in D^OTHERS^.

### ‘HDD 15.5 C (clim) [degrees C×days]’ [integer]

HDD calculated for a base temperature of 15.5 °C from D^clim^ at grid points closest to the lat/lon coordinates above, averaged over the same five-year period (2007–2011) as for the climate indices in D^GEA+^. HDD 15.5 C is defined as the sum over one year of the difference between 15.5 °C (base temperature) and the daily temperature, whenever the daily temperature is lower than the base temperature.

We noted that our computed HDD 15.5 C had poor correspondence (30–90%) with observations-based HDD 15.5 C in D^GEA+^ for 25 cities out of 56 cities with climate indices in D^GEA+^. Twelve cities are colder than predicted by the global model, and 5 of these cities are at high altitudes (>1000 m). The relatively course resolution of the ERA-interm dataset may not account for coastal and orographic effects^25^; this could lead to the prediction of fewer colder days than for the same coordinates but at higher altitude. The remaining 7 cities are at lower altitudes (13–935 m) and thus altitude effects likely do not explain why they are colder than the model predictions.

### ‘CDD 23 C (clim) [degrees C×days]’ [integer]

CDD calculated for a baseline temperature of 23 °C from D^clim^ at grid points closest to the lat/lon coordinates, averaged over the same five-year period (2007–2011) as for the climate indices in D^GEA+^. CDD 23 C is defined as the sum over one year of the difference between 23 °C (base temperature) and the daily temperature, whenever the daily temperature is higher than the base temperature.

We noted that our computed CDD 23 C had poor correspondence (30–90%) with observations-based CDD 23 C in D^GEA+^ for 53 cities out of 56 cities with climate indices in D^GEA+^. Forty-eight cities were warmer than predicted by the global model except for four cities (Bogotá, Mexico City, San Diego, and San Francisco), perhaps attributable to the urban heat island effect.

### 9. D^UEX^ Urban Area Expansion

D^UEX^ contains three types of urban area expansion attributes for two built-up areas (BUA) values (low and high) pertaining to three different years: 1990, 2000, and 2014. This results in 3 types×2 BUAs×3 years = 18 D^UEX^ attributes. The three types of attributes are BUAs [km^2^], BUA fraction [%] out of the total city area, and BUA population density [people/km^2^], as described below. The methodology for the preparation of D^UEX^, completed beforehand using ArcGIS (www.arcgis.com) and R using the city coordinates in D^clim^, is as follows:

#### Define area of interest (AOI) for each city: Urban Area Cluster (UAC)

The UAC is the study area for each city. The selection is based on city coordinates described above for D^clim^. UACs are approximations, reflecting estimates of urban catchment areas and do not depict administrative borders. UACs are selected based on the global human settlement (GHS) dataset (http://data.europa.eu/89h/jrc-ghsl-ghs_smod_pop_globe_r2016a). GHS is classified into high- and low-density and rural clusters, defined as:

*High-density clusters (HDCs)* comprise “contiguous cells (4-cell connectivity, gap filling) with a density of at least 1,500 inhabitants/km^2^ or a built-up density greater than 50%, and a minimum of 50,000 inhabitants per cluster”^30^.

*Low-density clusters (LDCs)* comprise “contiguous cells (4-connectivity, gap filling) with a density of at least 300 inhabitant/km^2^ and minimum of 5,000 inhabitants per cluster”^30^.

*Rural clusters* include all cells outside high- and low-density clusters^30^. From these areas, only the areas that intersect with one of the leftover city points are selected.

In the first round, contiguous areas from the HDC that intersect with one of the city points were identified and extracted. In the second round, contiguous LDC areas were identified and extracted for the city points that did not intersect with HDC in the first round. Note: two cities in D^CDP^ (Ærøskøbing, Denmark and Village of Kadiovacik, Turkey) are classified as rural and therefore were not considered in this analysis.

A buffer was applied, whereby the buffer distance was chosen based on the size of the urban cluster (classification “Jenks Natural Breaks”). Classes (m^2^) within the following ranges: 8,000,000–100,000,000; 100,000,001–300,000,000; 300,000,001–2,000,000,000; 2,000,000,001–4,000,000,000; 4,000,000,001–6,015,000,000 were assigned buffer distances (km) of 3, 5, 7, 10, and 13, respectively.

The results are the UACs for each city point. Importantly, some city points fell into the same UAC since these cities are close together and these settlements are contiguously connected. There are up to 9 city points in one cluster, as in e.g. the San Francisco bay area. In these cases, the standard buffer process was used nonetheless, including neighbouring urban areas, for methodological consistency. There are 343 city points but only 282 urban area clusters.

#### Classification of built-up areas into low- or high-density areas

Built-up intensity changes for each UAC were calculated based on built-up area raster grids (GHG BUILT-UP_GRID) (http://data.europa.eu/89h/jrc-ghsl-ghs_built_ldsmt_globe_r2015b). Datasets were classified into ‘low’ and ‘high’ for values between 0–25% and >25% of the built-up area (BUA), respectively, for the years 1990, 2000 and 2014. Classified raster data were added up for the change analysis and the areas calculated for high and low BUA.

#### Changes in population density (population intensification/sprawl)

Population densities are extracted (zonal statistics) within the classified built-up areas, high and low, for the 1990 baseline. Changes are captured from 1990–2000 and from 2000–2015. Note that the population raster dataset (GPWv4) (http://data.europa.eu/89h/jrc-ghsl-ghs_pop_gpw4_globe_r2015a) depicts the 2015 population, whereas the latest built-up area layer is from 2014. The 18 attributes obtained from D^UEX^ for 282 urban clusters corresponding to the 343 cities in D^final^ are described below:

### ‘Urban area name (UEX)’ [string]

Name of the urban area cluster.

### ‘Low BUA – *year* (UEX) [km2]’ [integer]

Low Built-up Area (BUA) for *year* = 1990, 2000, and 2014; three attributes in total.

### ‘High BUA – *year* (UEX) [km2]’ [integer]

High BUA for *year* = 1990, 2000, and 2014; three attributes in total.

### ‘Low BUA % – *year* (UEX) [percent]’ [integer]

Percentage of low BUA of the total BUA for *year* = 1990, 2000, and 2014, where total BUA is the sum of low and high BUA; three attributes in total.

### ‘High BUA % – *year* (UEX) [percent]’ [integer]

Percentage of high BUA of the total BUA for *year* = 1990, 2000, and 2014, where total BUA is the sum of low and high BUA; three attributes in total.

### ‘Low BUA population density – *year* (UEX) [people/km2]’ [integer]

The population density in the low BUA for *year* = 1990, 2000, and 2014; three attributes in total.

### ‘High BUA population density – *year* (UEX) [people/km2]’ [integer]

The population density in the high BUA for *year* = 1990, 2000, and 2014; three attributes in total.

### 10. D^OTHERS^ dataset

The attributes obtained from other external sources are as follows:

### ‘Latitude (others) [degrees]’ [decimal format]

City latitude. Used for HDD/CDD calculations.

### ‘Longitude (others) [degrees]’ [decimal format]

City longitude (decimal format). Used for HDD/CDD calculations.

### ‘Coordinate source (others)’ [string]

Source of city coordinates: GeoHack (https://www.mediawiki.org/wiki/GeoHack), obtained from the city’s Wikipedia page.

### ‘Population (others)’ [integer]

Population values obtained from external sources, consistent with the population and city definition in D^emissions^ where possible. We noted the largest discrepancies (30–90%) in population values in D^OTHERS^ vs. D^CDP^ in three cities:

*Brasília*: The population value in D^CDP^ was 1,409,671 (2015 estimate) vs. 2,977,216 (2016 estimate) in D^OTHERS^ (https://en.wikipedia.org/wiki/Bras%C3%ADlia#cite_note-pop-1). The IBGE average household size value from the 2010 census^31^ also corresponds to a population of 2,977,216.

*Caracas*: The population value in D^CDP^ was 3,518,590 (2015 estimate) vs. 5,290,000 (2016 estimate) in D^OTHERS^ (http://www.c40.org/cities/caracas, https://en.wikipedia.org/wiki/Metropolitan_Region_of_Caracas).

*Santiago de Guayaquil*: The population value in D^CDP^ was 2,350,915 (2010 estimate) vs. 3,500,000 (2014 estimate) in D^OTHERS^ (https://en.wikipedia.org/wiki/Guayaquil).

### ‘Population year (others)’ [integer]

Year corresponding to the external population values.

### ‘Population source (others)’ [string]

Source for external population values.

### ‘City area (others) [km2]’ [float]

City area obtained from external sources, consistent with population, area and city definition in D^emissions^.

### ‘City area source year (others)’ [integer]

Year corresponding to the area value.

### ‘City area source (others)’ [string]

Source for area value.

### ‘Diesel price 2014 (others) [USD/liter]’ [float]

2014 diesel price, from national values^22^.

### ‘Gasoline price 2014 (others) [USD/liter]’ [float]

2014 gasoline price, from national values^22^.

### ‘Household size (others) [people/household]’ [float]

Average number of people per household obtained from multiple sources. All updated household size values we collected were obtained for populations that matched the city population in D^emissions^. Note that the following assumptions were made to estimate household size in the cities listed below:

*Addis Ababa*: The 1984 value for average household size^32^ was used since a more recent value could not be found.

*Aspen and Pitkin county*: Average household size for “Aspen and Pitkin county” (population 7,710) was assumed to be close enough to household size of “Aspen” (population 6,871), since population values are close (https://www.census.gov/quickfacts/fact/table/aspencitycolorado/PST045216).

*Bogor*: The average household size of West Java in 2012 survey report^33^ was assumed to be representative of the average household size of Bogor, a city in West Java, since a value for Bogor itself could not be found. (As a check, this survey also reported a household size for East Jakarta (4.4) which is consistent with the value obtained for a 2014 Jakarta survey^34^ (4.5).)

*Bornova*: The average household size of Izmir province found in the 2011 census^35^ was used for Bornova.

*Caracas*: The population of Caracas in D^CDP^ (3,518,590) corresponds to the population of the Metropolitan Region of Caracas (https://en.wikipedia.org/wiki/Metropolitan_Region_of_Caracas) which is made up of the Metropolitan District of Caracas (=Distrito Capital + 4 Miranda state municipalities) plus 11 municipalities over Miranda and Vargas states. Therefore, to estimate household size, the household sizes of Distrito Capital, Miranda and Vargas states were averaged: 1/3×(3.41 + 3.52 + 3.58) = 3.50.

*Durban*: The population of Durban in D^CDP^ is 3,555,868, but actually this corresponds to the population of eThekwini Metropolitan Municipality (see https://en.wikipedia.org/wiki/EThekwini_Metropolitan_Municipality and

http://www.statssa.gov.za/?page_id=993&id=ethekwini-municipality). Therefore, the average household size for eThekwini (3.4, http://www.statssa.gov.za/?page_id=993&id=ethekwini-municipality) and not Durban (2.8, http://www.statssa.gov.za/?page_id=4286&id=10350) was used.

*Incheon*: Average household size in the 2015 census report^36^ is stated as 2.65 for “Incheon Province”; this value was assumed to be valid for Incheon city.

*Jakarta*: Average household size for Jakarta was based on a survey^34^ of 297 households in 2014.

*Pretoria Tshwane*: The most recent household size statistic that could be found (3.40)^37^ corresponds to year 2007 and has a similar value as in the original D^GEA+^.

*Quito and Santiago de Guayaquil*: The 1990 value for average household size^38^ was used since a more recent value could not be found. Note also that this value is listed as “Average number of persons per living quarters”.

*Suwon*: Only the average household size for Gyeonggi province (2.68) could be found^36^. Since Suwon is the largest metropolis of Gyeonggi province, this value was assumed to be representative of the average household size in Suwon.

*Santiago de Cali*: The average household size for Valle de Cauca in the 2005 census^39^ was used. Santiago de Cali is the capital of the Valle del Cauca department, and the most populous city in southwest Colombia.

### ‘Household size year (others)’ [integer]

Year for which the mean household size above is given.

### ‘Household size source (others)’ [string]

Source reference for updated household sizes.

### ‘Household size comment (others)’ [string]

Updated comments pertaining to household size. For example, the original comment in D^GEA+^ for city ‘Birmingham’ was “Calculation: pop/household (1036900/410700)”. The updated household size for Birmingham, however, was obtained directly from EUROSTAT (http://ec.europa.eu/eurostat/) without the need for calculation; therefore, this comment was removed.

### ‘Mean one-way travel time (others) [minutes]’ [float]

Mean one-way travel time to travel to work for city citizens. All values we collected were obtained for populations that matched the city population in D^emissions^. The following assumptions that were made in estimating mean commute time for some cities.

*Ajax, ON*: The mean commute time for Durham region was used since a value could not be found for the city of Ajax, Ontario.

*Birmingham*: The mean commute time was taken from Telegraph article (http://www.telegraph.co.uk/motoring/news/3122428/Birmingham-worst-place-for-commuting-survey.html and https://www.findaphd.com/search/projectdetails.aspx?PJID=61628), but the original reference could not be found.

*Leicester, Manchester*: The mean commute time was taken from Telegraph article (http://www.telegraph.co.uk/motoring/news/3122428/Birmingham-worst-place-for-commuting-survey.html and https://www.findaphd.com/search/projectdetails.aspx?PJID=61628), but the original reference could not be found.

*Seoul*: Two sources for mean commute time were found: one value (41.2 min) from the 2010 Statistics Korea census referenced by a 2011 KBS World Radio article (http://world.kbs.co.kr/english/news/news_Ec_detail.htm?No=84738), and another value (48.0 min) from a “big data analysis conducted by the nation’s No. 2 mobile carrier KT” referenced in the a 2017 Korea Herald article (http://www.koreaherald.com/view.php?ud=20170621000809&mod=skb). The latter value was used since it is most recent of the two values.

*Suwon*: The reference above for Seoul also reports an average mean commute time (43.5 min) for Gyeonggi province. Since Suwon is the largest metropolis of Gyeonggi province, it was assumed that this was representative of Suwon.

*Rotterdam*: The average commute time for MRDH (Metropoolregio Rotterdam Den Haag) across education levels was used^40^. However, MRDH comprises 23 out of 60 municipalities in Zuid-Holland province, and its population and area are much larger than Rotterdam itself (2.24 M vs. 620 k, and 997 km^2^ vs. 208 km^2^, respectively).

*Mexico City*: The mean commute time (75 min) was obtained from a 2017 Mexico News Daily article (http://mexiconewsdaily.com/news/45-days-a-year-spent-commuting-in-cdmx/), but the original El Universal report cited could not be found. However, this value is consistent with the 61–90 minutes estimation in a 2008 report from the Universidad Nacional Autónoma de México^41^.

*Caracas, Lima*: mean travel time was based on 16 contributors only (www.numbeo.com).

*Ljubljana*: mean travel time was based on 30 contributors only (www.numbeo.com).

*Vlinius*: mean travel time was based on 46 contributors only (www.numbeo.com).

*Reykijavik*: mean travel time was based on 22 contributors only (www.numbeo.com).

*Athens*: mean travel time was based on 117 contributors only (www.numbeo.com).

*Buenos Aires*: mean travel time was based on 42 contributors only (www.numbeo.com).

*Adelaide*: mean travel time was based on 37 contributors only (www.numbeo.com).

*Canberra*: mean travel time was based on 31 contributors only (www.numbeo.com).

*Melbourne*: mean travel time was based on 81 contributors only (www.numbeo.com).

*Santiago*: mean travel time was based on 36 contributors only (www.numbeo.com).

*Hong Kong*: mean travel time was based on 53 contributors only (www.numbeo.com).

*Copenhagen*: mean travel time was based on 49 contributors only (www.numbeo.com).

*Torino*: mean travel time was based on 23 contributors only (www.numbeo.com).

*Amman*: mean travel time was based on 25 contributors only (www.numbeo.com).

*Warsaw*: mean travel time was based on 83 contributors only (www.numbeo.com).

*Cape Town*: mean travel time was based on 74 contributors only (www.numbeo.com).

*Durban*: mean travel time was based on 17 contributors only (www.numbeo.com).

*Johannesburg*: mean travel time was based on 81 contributors only (www.numbeo.com).

*Pretoria Tshwane*: mean travel time was based on 21 contributors only (www.numbeo.com).

*Taipei City*: mean travel time was based on 26 contributors only (www.numbeo.com)

*Quito*: calculated based on the following estimation based on four time windows weighted by the fraction of time spent for each^38^:

0.2×(15 min/2) + 0.39×(15 min + 7.5 min) + 0.24×(30 min + 15 min) + 0.08×(60 min + 30 min)

### ‘Mean one-way travel time year (others)’ [integer]

Year for which mean travel time to work is given.

### ‘Mean one-way travel time source (others)’ [string]

Source for mean travel time values.

### GDP

D^final^ includes GDP values at Purchasing Paring Parity (GDP-PPP) and nominal GDP (nGDP) obtained from multiple GDP assessments, as specified in the ‘source’ columns. Altogether, these references gap-filled the GDP-PPP attribute for 153 cities (96 cities in D^CDP^, 17 cities in D^carbonn^, and 40 cities in D^PKU^), and gap-filled the nGDP attribute for 211 cities (116 cities in D^CDP^, 25 cities in D^carbonn^, and 70 cities in D^PKU^).

### ‘GDP-PPP (others) [$BN]’ [float]

GDP-PPP values in billions USD.

### ‘GDP-PPP/capita (others) [USD/capita]’ [float]

GDP-PPP normalized by population values referenced in the same source.

### ‘GDP-PPP source (others)’ [string]

The following sources were used to obtain GDP-PPP in the order listed below:

Brookings Institution for 2014 (https://www.brookings.edu/research/global-metro-monitor); 140 cities matched D^final^. The original units [$Million] were converted to [$Billion] for consistency with other GDP datasets. The population values were obtained from the same data source for the same reference year (2014).

Brookings Institution for 2015 (https://www.brookings.edu/research/redefining-global-cities/#cancel); 5 cities matched D^final^. The population values were obtained from the same data source for the same reference year (2015).

PricewaterhouseCoopers (PwC) for the year 2008 (originally given with projections to 2025) Report (https://web.archive.org/web/20110504031739/https://www.ukmediacentre.pwc.com/imagelibrary/downloadMedia.ashx?MediaDetailsID=1562), obtained for 8 cities in D^final^ for which a value from Brookings Institution for 2014 was not available.

### ‘GDP-PPP year (others)’ [integer]

Year corresponding to the GDP-PPP value.

### ‘nGDP (others) [$BN]’ [float]

Values for nominal GDP (nGDP; GDP estimates at current market prices) in billions USD.

### ‘nGDP/capita (others) [USD/capita]’ [float]’

Nominal GDP normalized by population values referenced in the same source.

### ‘nGDP source (others)’ [string]

The following sources were used to obtain nGDP in the order listed below:

McKinsey for the year 2010 (http://foreignpolicy.com/2012/08/07/the-most-dynamic-cities-of-2025/, or displayed as an interactive map http://www.mckinsey.com/tools/Wrappers/Redesign/InteractiveWrapper.aspx?sc_itemid={C84CB74F-A3B1-47B1-8265-6252F6D85B68}), of which 168 cities matched D^final^.

The Organisation for Economic Co-Operation and Development (OECD) (available at https://stats.oecd.org/). The OECD GDP values were found for 27 cities in D^final^ that did not have a value from McKinsey 2010.

Various external sources, principally from Wikipedia (https://en.wikipedia.org/wiki/List_of_cities_by_GDP#cite_ref-42), found for 14 cities that did not have a value from McKinsey 2010 or from OECD.

### ‘nGDP year (others)’ [integer]

Year corresponding to the nGDP value.

### ‘Exports (others) [m3]’ [integer]

Domestic natural gas export.

### ‘Production (others) [m3]’ [integer]

Domestic natural gas production.

### ‘Natgas Export/Production ratio (others)’ [float]

Ratio of domestic natural gas export to production.

### ‘CH4_waste/capita [tCH4/capita] (others)’ [float]

National per capita methane waste emissions multiplied by city population using country emissions data and population from the EDGAR inventory for year 2010 (Data Citation 1).

### ‘CH4_waste+natgas/capita [tCH4/capita] (others)’ [float]

National per capita methane emissions from waste and natural gas production multiplied by city population using country emissions data from the EDGAR inventory for year 2010 (Data Citation 1) and population data from the World Factbook (2010 values, https://www.cia.gov/library/publications/the-world-factbook/rankorder/rankorderguide.html).

### ‘Corrected CH4_(waste+natgas)/capita (others) [tCH4/capita]’ [float]

National methane emissions from natural gas production scaled to account for import and export.

### ‘Year from CIA (others)’ [integer]

Year of national natural gas export data published in the CIA World Fact Book 2014 (https://www.cia.gov/Library/publications/the-world-factbook/geos/bm.html).

## Technical Validation

Technical validation includes analyses to support the technical quality of the dataset. Five technical validations were performed. Firstly, a calculation of quality flags for assessing the consistency of city area between different datasets merged in D^final^; secondly, the temporal consistency between emissions, population and household size data (see Table 5). Thirdly, the calculation of quality flags for the consistency of Scope-1 CO_2_ emission data within D^CDP^ (see Table 3). Finally, a comparison of emissions data between D^final^ vs. VULCAN^42^, D^final^ vs. Markolf *et al.*^43^ for US cities, and D^final^ vs. Nakamichi *et al.*^44^ for six Japanese cities.

### 1. Quality Flags for area consistency between datasets

City area (spatial unit) differs between datasets, which led us to define an area quality flag (AQF). Differences of the year for which population household size and Scope-1 CO_2_ emissions were estimated led us to define temporal quality flags, for population (PQF) and household size (HQF), respectively.

### ‘AQF (CDP/GEA)’ [integer; value 0 or 1]

Determined from the ratio of city areas of D^CDP^ to D^GEA^ (attributes ‘City area (CDP) [km2]’ and ‘City area (GEA) [km2]’), AQF (CDP/GEA) = 1 is set for ratios within range [0.5, 2] and AQF (CDP/GEA) = 0 otherwise. Inconsistent areas between D^CDP^ and D^GEA^ with AQF(CDP/GEA) = 0 were found for 10 out of 30 common cities. Four inconsistencies were attributable to different city boundary definitions:

*Stockholm*: D^CDP^ area is for ‘City of Stockholm’, while D^GEA^ area is for ‘Stockholm County’.

*Toronto*: D^CDP^ area is for ‘City of Toronto’, while D^GEA^ area is for ‘Greater Toronto Area’.

*Cardiff*: D^CDP^ area is for ‘City of Cardiff’, while D^GEA^ area is for ‘Cardiff and Vale of Glamorgan’.

*Birmingham*: D^CDP^ area is for ‘Birhmingham City Council’, but city boundary is not specified in D^GEA^.

Similarly to AQF (CDP/GEA), other AQF (X/Y) flags were computed for cities areas across pairs of datasets (X/Y), namely D^CDP^ and D^WB^, D^CDP^ and D^OTHERS^, D^PKU^ and D^GEA^, D^PKU^ and D^GEA^, and D^PKU^ and D^OTHERS^.

### ‘AQF (CDP/WB)’ [integer; value 0 or 1]

From the ratio of areas in D^CDP^ to D^WB^, AQF (CDP/WB) = 0 were found for 3 out of 15 cities. Two inconsistencies are attributable to different city boundary definitions and a data entry error:

*Los Angeles*: D^CDP^ is for ‘City of Los Angeles’ while D^WB^ for ‘Los Angeles County’.

*Toronto*: D^CDP^ is for ‘City of Toronto’ whereas D^WB^ is for ‘Greater Toronto Area’.

*Seattle*: the D^CDP^ area value was a typo (D^WB^ and D^OTHERS^ area values are consistent).

### ‘AQF (CDP/OTHERS)’ [integer; value 0 or 1]

From the ratio of areas in D^CDP^ vs. D^OTHERS^ areas, AQF (CDP/OTHERS) = 0, were found in 19 out of 184 cities. Areas were not reported for four D^CDP^ cities (Aarhus Kommune, Bornova, Palmas). Three data entry errors in D^CDP^ areas were found and corrected for Faro, Porto Alegre, Salvador, and manually corrected in D^final^. The D^CDP^ area of Kadiovacik is likely not 5,947 km^2^ as reported and was estimated manually to be 0.23 km^2^ by drawing a square around its center using https://www.daftlogic.com/projects-google-maps-area-calculator-tool.htm). It is unclear if remaining area inconsistencies were due to different city boundary definitions or e.g. data entry errors.

### ‘AQF (PKU/GEA)’ [integer; value 0 or 1]

From the ratio of areas in D^PKU^ vs. D^GEA^, AQF (PKU/GEA) = 0 were found for all 19 common cities. The built-up area reported in D^PKU^ was consistently much smaller than the area reported in D^GEA^, likely due to city boundary definitions.

### ‘AQF (PKU/WB)’ [integer; value 0 or 1]

From the ratio of areas in D^PKU^ vs. D^WB^ area, AQF (PKU/WB) = 0 were found for all 3 common cities. The built-up area reported in D^PKU^ is consistently much smaller than in D^WB^, likely due to city boundary definitions.

### ‘AQF (PKU/OTHERS)’ [integer; value 0 or 1]

From the ratio of areas in D^PKU^ vs. D^OTHERS^ area, AQF (PKU/others) = 0, were found for 73 out of 77 cities. The built-up area reported in D^PKU^ is consistently much smaller than the area reported in D^OTHERS^, likely due to city boundary definitions.

### 2. Quality Flags for temporal consistency between variables

Temporal consistency means similarity of the year for which population household size and Scope-1 CO_2_ emissions were estimated, which defines temporal quality flags for population (PQF) and household size (HQF).

### ‘PQF (CDP)’ [integer; value 0 or 1]

Determined from the absolute difference between the year of Scope-1 emissions in D^emissions^ (in this case, CDP emissions) and the year of D^CDP^ population. TQF value = 1 for differences less than 5 years, and 0 otherwise. Inconsistent values were found for 14 out of 187 cities. Most of these inconsistencies were on or near the cutoff 5-year point (5 years: 8 cities, 7 years: 2 cities, 8 years: 1 city, 11 years: 1 city; range 5–24 years). The remaining two cities (Piacenza, Winnipeg) have recent population years (2014 and 2015, respectively) but not very recent emission years (1990 and 1998, respectively).

### ‘PQF (carbonn)’ [integer; value 0 or 1]

Inconsistent values were found for 11 out of 68 cities. Most of these inconsistencies were on or near the cutoff 5-year point (5 years: 3 cities, 6 years: 4 cities, 7 years: 1 city, 8 years: 1 city, 9 years: 1 city; range 5–20 years). The remaining city (Graz) has a recent population year (2014) but a quite out-dated emission year (1994).

### ‘PQF (WB)’ [integer; value 0 or 1]

Inconsistent values were found for 17 out of 20 cities. In these cases, the difference in population and emission years ranged from 6–15 years.

### ‘PQF (WB2010)’ [integer; value 0 or 1]

Note that this PQF was determined from the 2010 population value in D^WB^ (attribute ‘Population 2010 (WB)’ in D^final^). Inconsistent values were found for 2 out of 20 cities. In these cases, the difference in population and emission years was on the cutoff point of 5 years.

### ‘PQF (OTHERS)’ [integer; value 0 or 1]

Inconsistent values were found for 59 out of 269 cities in D^OTHERS^ (difference range 5–22 years).

### ‘HQF (GEA+)’ [integer; value 0 or 1]

Determined from the absolute difference between the year of Scope-1 emissions in D^emissions^ and the year of D^GEA+^ household size, where these values exist. HQF value = 1 for differences less than 15 years (given the slower changes in this variable), and 0 otherwise. No inconsistent HQF (GEA+) values were found for all 103 cities for which household size values exist in D^GEA+^.

### ‘HQF (OTHERS)’ [integer; value 0 or 1]

Defined as in HQF (GEA+) above but for household sizes in D^OTHERS^. Inconsistent values were found for two out of 176 cities for which external household size values were obtained (Graz and Piacenza). These inconsistencies are due to quite out-dated emission years [1994 for Graz (D^carbonn^) and 1990 for Piacenza (D^CDP^)].

### 3. Quality Flags for D^CDP^ emissions

In this section, we analyzed the technical quality of the CDP dataset and created four types of Quality Flags for emissions in D^CDP^ (see Table 3).

### Emissions Quality Flag

Emission Quality Flags (EQF) were computed for D^CDP^ emissions based on a sequential series of checks assessing the consistency between reported total emissions (TOT) and scope-specific values (S1, S2) as shown in Figure 3. Four cases were possible for EQF: TOT = S1 or S2; TOT ≈ S1 + S2 (within±15%); TOT ≠ S1 + S2; either S1 or S2 or both values missing. Where TOT = S1 or S2 and emissions of both scopes existed, TOT was recalculated by summing the two scopes. Where S2 was missing, S1 was inferred by subtracting the existing scope data from TOT. In all other cases that failed the QA/QC, D^CDP2016^ values were replaced by D^CDP2017^ values if the latter had a better quality flag.

EQF = A, B, C, D, or E were assigned based on the steps shown in Fig. 3. EQF = A was applied to emissions that satisfied TOT ≈ S1 + S2 (32 cities) or TOT = S1 + S2 (86 cities) or where TOT = S1 or S2 and could therefore be recalculated by summing the scopes (4 cities); EQF = B was applied to cities where TOT = S1 + S2 and Scope 3 was said to be reported in TOT, which cannot both be true (11 cities); EQF = C was applied to emissions in which TOT and S1 existed (3 cities) or TOT and S2 existed (6 cities; S1 then derived from TOT − S2). EQF = D was assigned to cities missing both scopes (35 cities), and EQF = E was assigned to cities where S1 is likely correct, S2 = 0, and TOT = S1 + S2 = S1 (7 cities). In total, the QA/QC procedure led us to replace emissions from 34 cities in D^CDP2016^ by those from D^CDP2017^, and to have EQF = D for 36 cities missing both scopes. Special case for Rotterdam: D^CDP2016^ data was EQF = A, but included emissions from several facilities in the port and were replaced by D^CDP2017^ values (EQF = C, S2 missing).

### 4. Validation of Scope-1 CO_2_ emissions against VULCAN

The uncertainty of reported emissions from cities is particularly difficult to estimate since no formal uncertainty analysis is applied by cities on the bottom-up activity and emission factor data that they collect for inventories. In our processing of D^CDP^ emission data, systematic errors were also introduced when removing non-CO_2_ gases emissions. Further, whether Scope-1 data from D^CDP^, and D^carbonn^ and D^PKU^ included all power plants in the territory of each city was not verified systematically against independent sources. Many of these city power plants can be small and may not be reported by each city.

Accurate estimates for city-scale emissions uncertainties depends on a clear understanding of system boundaries, i.e. emitting activities included in the accounting for Scope-1. A large source of errors between city emission estimates is the use of distinct systems boundaries (e.g. counting or not cement manufacturing, industrial sites, small power plants, biofuels). Once these inconsistencies are corrected, the between-estimates uncertainty could be reduced.

A detailed evaluation against independent estimates is arguably the best practical way to assess uncertainties when looking at a multiple-cities dataset. This is the approach we followed in this section.

The VULCAN dataset from Gurney *et al.*^42^ provides U.S. fossil fuel CO_2_ emissions for the year 2002 on a 10 × 10 km grid at the level of fuel type, economic sub-sector, and county/state identification over the entire US landscape. VULCAN includes individual factories, power plants, roadways and neighborhoods. To compare with our Scope-1 values for US cities, we considered two scenarios: 1) summed VULCAN emissions from all the sectors of aircraft, cement, commercial, industrial, non-road, on-road, residential, and electricity production and 2) we removed from the sum of all-sectors the emissions from electricity production. Emissions at the location of 64 US cities in D^final^ (63 from D^CDP^ and 1 from D^carbonn^) were collocated with the gridded VULCAN data, assuming that each city is approximately a circle of area corresponding to the reported administrative area in our dataset. Scope-1 emissions and Scope-1 emission density (emissions/area) in D^final^ were regressed against the two corresponding VULCAN scenarios including and excluding emissions from electricity production. The correlation coefficient (R^2^) when electricity production emissions were included was 0.69 (respectively 0.70 when excluded) for Scope-1 emissions and 0.56 (0.59) for Scope-1 emission density. The lower correlation coefficient in emission density for both cases is due to the coarse approximation of the city shape (using circles for cities areas instead of detailed GIS administrative area shape files overlaid with VULCAN gridded data) and the low resolution of VULCAN, and/or the different years they represent.

We also calculated the median absolute deviation (MAD) between VULCAN and D^final^ Scope-1 emissions. MAD = 99% and 82%, including and excluding power plant electricity generation, respectively. Most of this error is of a systematic nature due to the following reasons: emissions are for year 2002 in VULCAN and for more recent years in D^final^, the VULCAN gridded data were not sampled for the precise administrative area of D^final^ in each US city, we lack precise knowledge on whether all local power plant emissions were included in Scope-1 data in D^final^ as compared to the comprehensive set of city power plants included in VULCAN. Scope-1 emissions of at least 6 out of 14 US cities in D^final^ include power plants; but 20 cities in D^final^ reported zero within-city power plant emissions in their Scope-1 emissions. Other cities likely reported power plants but we could not verify it from their declarations.

### 5. Validation of Scope-1 CO_2_ emissions per capita against Markolf *et al.*

The second dataset against which we compared Scope-1 CO_2_ emissions in D^final^ for US cities is described in Markolf *et al.*^43^. The authors compiled publicly available national datasets for estimating emissions in the 100 most populated metropolitan areas in the US in 2014. Twenty-eight cities in Markolf *et al.* overlapped with D^final^. Markolf *et al.* documented separately CO_2_ emissions from on-road transportation, electricity generation, industrial processes, residential buildings, commercial buildings and waste. Then, the emissions were divided by population because the metropolitan areas in Markolf *et al.* generally encompass a wider territory than in D^final^. The ratio between per capita Scope-1 emissions of Markolf *et al.* to D^final^ was calculated, both including and excluding electricity generation in Markolf *et al.* When electricity generation was excluded, there were three outliers with a ratio ≥2 (*Houston*, ratio 2.6; *New Orleans*, ratio 4.6; *Indianapolis*, ratio 12.7); the mean ratio excluding these outliers was 1.2 ± 0.4. When electricity generation was included, there were 11 outliers with ratios ≥2; the mean ratio excluding these outliers was 1.3 ± 0.4.

The MAD statistics between Markolf and D^final^ per-capita Scope-1 emissions for the 28 common US cities is 393% and 175% (including and excluding power plant electricity generation, respectively). These ratios were reduced when the outliers (see above) were removed, giving MAD of 197% and 161% (including and excluding power plant electricity generation, respectively). We note that some of the inventory data used by Markolf *et al.* may be common with D^CDP^ and D^carbonn^, thus Markolf *et al.* cannot be proven to be strictly independent from our data.

### 6. Validation of Scope-1 CO_2_ emissions against Nakamichi *et al.*

The third dataset against which we compared D^final^ Scope-1 CO_2_ emissions was developed for all municipalities in Japan and described in Nakamichi *et al.*^44^ The authors computed direct CO_2_ emissions using a bottom-up (built-up) approach based on emission factors and other statistics attributed to four civilian categories: residential, commercial, industrial (including electricity production) and transportation, as well as the total integrated direct emissions. The year of analysis was 2005 for all categories. Six cities in Nakamichi *et al.* overlapped with D^final^. The ratio between Scope-1 emissions of Nakamichi *et al.* to D^final^ was calculated, taking into account the ratios between reported population and city areas, which are in agreement (the ratio CDP/Nakamichi *et al.* or carbonn/Nakamichi *et al.* is in the range 1.0–1.1). The mean Scope-1 emission ratio was 0.8 ± 0.4 (range 0.4–1.3). Excluding Tokyo, corresponding to the worst ratio (0.4) despite consistent population and area ratios, the mean Scope-1 emission ratio was 0.9 ± 0.3 (range 0.5–1.3).

## Additional information

**How to cite this article**: Nangini, C. *et al*. A global dataset of CO_2_ emissions and ancillary data related to emissions for 343 cities. *Sci. Data*. 6:180280 doi: 10.1038/sdata.2018.280 (2019).

**Publisher’s note**: Springer Nature remains neutral with regard to jurisdictional claims in published maps and institutional affiliations.

## Supplementary Material



## Figures and Tables

**Table 1:** Datasets and their corresponding method steps.

Datasets D*	Description	Step in Methods where D* is handled/created
D^CDP2016^, D^CDP2017^ = > D^CDP^	CDP Scope-1 CO_2_ emissions calculated from the 2016 and 2017 CDP datasets.	Step 1
D^CH4^	Previously created dataset containing values for estimating CO_2_ from CO_2-eq_.	Step 2
D^CDP^	Estimation of CO_2_ emissions from CO_2-_eq	Step 2
D^carbonn^	Scope-1 emissions from carbon*n*	Step 3
D^PKU^	Scope-1 emissions from the Chinese dataset	Step 3
D^GEA^, D^UITP^, D^WB^=> D^GEA+^	Published ancillary data from GEA, UITP, and WB, respectively	Step 4
D^OTHERS^	Other ancillary data obtained from external sources	Step 4
D^INRIX^	Previously-prepared INRIX traffic-related data	Step 4
D^TomTom^	Previously-prepared TomTom traffic-related data	Step 4
D^IESE^	Previously-prepared IESE socio-economic indicators	Step 4
D^UEX^	Calculated urban expansion data	Step 4
D^clim^	Previously-calculated climate indices	Step 4
D^final^	Final dataset	
Datasets D*, their description and the Method Section steps where they are handled/created.		

**Table 2:** City attributes.

D^final^ attribute	D*	# CDP cities					Reference(s) cited in D*
*Descriptive info*		*orig*	*final*				
'City Name'	—	—	343	
'City Name (CDP)'	D^CDP^	187	—	provided by city
'City Name (carbonn)'	D^carbonn^	73	—	provided by city
'City Name (PKU)'	D^PKU^	83	—	provided by source
'City Name (GEA)'	D^GEA^	97	—	provided by source
'City Name (UITP)'	D^UITP^	49	—	provided by source
'City Name (WB)'	D^WB^	20	—	provided by source
'Definition (CDP)'	D^CDP^	187	—	provided by city
'Definition (carbonn)'	D^carbonn^	73	—	provided by source
'Definition (WB)'	D^WB^	15	—	provided by source
'Study Year (WB)'	D^WB^	15	—	provided by source
'City location (CDP)'	D^CDP^	187	—	provided by source
'Latitude’, ‘Longitude’, ‘Coordinate source’	D^clim^	343	—	GeoHack (https://www.mediawiki.org/wiki/GeoHack)
'Country'	—	343	—	Corresponding D* or added manually
'Region'	—	343	—	Based on categories in D^carbonn^
'Reporting year (CDP)'	D^CDP^	187	—	provided by city
'Ancillary from GEA+‘	D^GEA+^	117	—	Flags if attributes were added from D^GEA+^
***Population, area, density***				
'Population'	D^CDP^	186	186	provided by city
'Population year (CDP)'	D^CDP^	187	187	provided by city
'Population (carbonn)'	D^carbonn^	73	73	provided by source
'Population Year (carbonn)'	D^carbonn^	73	73	provided by source
'Urban Population (PKU)'	D^PKU^	83	83	provided by source
'Population (GEA)'	D^GEA^	41	41	provided by source
'Population (UITP)'	D^UITP^	42	42	provided by source
'Population (WB)’, ‘year’	D^WB^	15	15	provided by source
'Population (others)’, ‘year’, source’,	D^OTHERS^	—	343	Multiple sources. See D^final^.
'Population 1950 (WB)’, ‘Population 1990 (WB)’, ‘Population 2010 (WB)'	D^WB^	15	15	World Urbanization Prospects: The 2011 Revision; UN-Department of Economic and Social Affairs, Population Division
'Growth Rate 1950-2010’, ‘1990-2010’ (WB)	D^WB^	15	15	computed
'City area [km2] (CDP)'	D^CDP^	184	184	provided by city
'City area [km2] (others)'	D^OTHERS^	—	187	Multiple sources. See D^final^.
'City area [km2] (GEA)'	D^GEA^	30	30	Wikipedia value for definition, which corresponds to closest population value, incomplete
'City area [km2] (WB)'	D^WB^	15	15	Wikipedia values corresponding to definition of city
'Built-up area (km2) (PKU)'	D^PKU^	83	83	provided by source
'City area [km2] (others)’, ’year’, ‘source’	D^OTHERS^			Multiple sources. See D^final^.
'Population density (GEA)'	D^GEA^	41	41	computed
'Population density (UITP)'	D^UITP^	42	42	provided by source
'Population density (WB)'	D^WB^	15	15	computed
'Population/sqrt(area) (GEA)'	D^GEA^	30	30	computed
'Population/sqrt(area) (WB)'	D^WB^	15	15	Computed
***Urban are expansion***				
'Urban area name'	D^UEX^	—	282	GHS settlement grid, http://data.europa.eu/89h/jrc-ghsl-ghs_smod_pop_globe_r2016a
'Low/High BUA’ for year 1990, 2000, 2014	D^UEX^	—	282	These data contain a multitemporal information layer on built-up presence as derived from Landsat image collections (GLS1975, GLS1990, GLS2000, and ad-hoc Landsat 8 collection 2013/2014). Resolution used: 250 m. Variable name: GHS built-up grid, http://data.europa.eu/89h/jrc-ghsl-ghs_built_ldsmt_globe_r2015b.
'Low/High BUA %‘ for year 1990, 2000, 2014	D^UEX^	—	282	GHS built-up grid, http://data.europa.eu/89h/jrc-ghsl-ghs_built_ldsmt_globe_r2015b
'Low/High population density’ for year 1990, 2000, 2014	D^UEX^	—	282	Spatial raster dataset [http://ghsl.jrc.ec.europa.eu/ghs_bu_s1.php?] that depicts the distribution and density of population, expressed as the number of people per cell. Residential population estimates for target years 1975, 1990, 2000 and 2015 provided by CIESIN GPWv4 were disaggregated from census or administrative units to grid cells, informed by the distribution and density of built-up as mapped in the Global Human Settlement Layer (GHSL) global layer per corresponding epoch. Resolutions available: 250 m, 1 km. Variable name: GHS population grid, http://data.europa.eu/89h/jrc-ghsl-ghs_pop_gpw4_globe_r2015a
***Emissions- and climate-related***				
'CO2 emissions per capita'	D^PKU^	83	83	provided by source
'Scope-1’, ‘Scope-1 dataset’, ‘units’, ‘year’, ‘Emissions protocol’	D^CDP^, D^carbonn^, D^PKU^	187, 73,		
83	187, 73,			
83	provided by source			
'Gases included'	D^CDP^	187	187	provided by source
'Methodology details'	D^CDP^	133	133	provided by city
'Increase/Decrease from last year'	D^CDP^	166	166	provided by city
'Reason for increase/decrease in emissions'	D^CDP^	144	144	provided by city
'Scope-2'	D^CDP^	141	141	provided by source
'Total emissions'	D^CDP^	187	187	provided by city
'CDP2016 data edited'	D^CDP^	42	42	computed
'Emissions Quality Flag'	D^CDP^	187	187	computed
'S1 lower bound'	D^CDP^	187	187	computed
'S1 upper bound'	D^CDP^	187	187	computed
'S1 mean'	D^CDP^	187	187	computed
'TOT lower bound'	D^CDP^	187	187	computed
'TOT upper bound'	D^CDP^	187	187	computed
'TOT mean'	D^CDP^	187	187	computed
'Scope fraction'	D^CDP^	187	187	computed
'Exports [m3]’, ‘Production [m3]'	D^OTHERS^	—	187	Multiple sources. See D^final^.
'Natgas Export/Production ratio'	D^OTHERS^	—	187	computed
'CH4_waste/capita’, ‘CH4_waste+natgas/capita’, ‘Corrected CH4_(waste+natgas)/capita'	D^OTHERS^	—	187	computed
'Average altitude'	D^CDP^			
D^carbonn^	187	187	- provided by source (CDP) and Geospatial Information Authority of Japan (3 cities in carbonn)	
'Average annual temperature'	D^CDP^	177	177	provided by city
'Weather station ID'	D^GEA+^	56	56	www.degreedays.net (temperature data from www.wunderground.com)
'HDD 15.5 C’, ‘CDD 23 C'	D^GEA+^	56	56	Ibid.
'HDD 15.5 C’, ‘CDD 23 C'	D^CLIM^	343	343	Computed
***Traffic-related***				
'Congestion rank'	D^INRIX^	95	95	Ibid.
'Peak hours spent in congestion'	D^INRIX^	95	95	From a subset of “300 million different sources” – please see original report^23^
'INRIX congestion index'	D^INRIX^	95	95	Ibid.
'Average congestion rate'	D^INRIX^	95	95	Ibid.
'Congestion rank'	D^TomTom^	88	88	Ibid.
'Congestion level'	D^TomTom^	88	88	“All data is based on actual GPS measurements from TomTom’s historical traffic database. For some cities we use GPS data from our partners, such as AutoNavi.” (https://www.tomtom.com/en_gb/trafficindex/)
'Congestion change'	D^TomTom^	75	75	Ibid.
'Morning peak'	D^TomTom^	88	88	Ibid.
'Evening peak'	D^TomTom^	88	88	Ibid.
'Mean one-way travel time’, ‘year’, ‘source'	D^OTHERS^	–	152	Multiple sources. See D^final^.
***Socio-economic***				
'Household size’, ‘year’, ‘source'	D^GEA+^	48	48	Multiple sources. See D^final^.
'Household size’, ‘year’, ‘source'	D^OTHERS^	—	167	Multiple sources. See D^final^.
'Center of commerce index'	D^GEA+^	33	33	MasterCard Worldwide (2008) Worldwide Centers of Commerce Index, New York, MasterCard.
'Urbanization ratio'	D^GEA+^	57	57	United Nations, Department of Economic and Social Affairs, Population Division (2012). World Urbanization Prospects: The 2011 Revision, CD-ROM Edition.
'Water bounded'	D^GEA+^	117	117	derived visually from google maps by source
'Other bounded'	D^GEA+^	117	117	Ibid.
'Diesel’ & ‘gas price’ ‘Diesel’ & ‘gas price’ 2014	D^GEA+^, D^OTHERS^	57	57	Ref^21,22^. For Japan, http://www.enecho.meti.go.jp/en/reports
all attributes in D^IESE^	D^IESE^	—	85	Please see citations in source^24^
'GDP’, ‘unit’, ‘year’,‘source'	D^CDP^	130	130	provided by source
'GDP’, ‘unit’, ‘year'	D^carbonn^	48	48	provided by source
'nGDP'	D^carbonn^	73	73	provided by source
'GDP [10000 RMB]'	D^PKU^	83	83	provided by source
'Income per capita [RMB]'	D^PKU^	83	83	provided by source
'GDP-PPP [$BN]’, ‘source'	D^OTHERS^	—	153	Multiple sources. See D^final^.
'GDP-PPP/capita [USD]'	D^OTHERS^	—	106	computed
'nGDP [$BN]’, ‘year’, ‘source’	D^OTHERS^	—	211	Multiple sources. See D^final^.
'nGDP/capita [USD]'	D^OTHERS^	—	195	computed
'GDP-PPP/capita (GEA)'	D^GEA^	97	97	provided by source
'GDP-PPP/capita (UITP)'	D^UITP^	49	49	Ibid.
'GDP-PPP/capita (WB)'	D^WB^	19	19	Ibid.
***Energy use***				
'Total final consumption per capita'	D^GEA^	97	97	provided by source
'Energy per capita CO2'	D^WB^	20	20	provided by source
Descriptive city attributes in the final dataset, their source dataset, the number of cities having the attributes before and after all data processing steps, and the reference(s) cited in the source dataset.				

**Table 3:** Emissions quality flags.

Emissions Quality Flag (EQF)	Definition	Number of D^CDP^ cities
A	TOT = S1 + S2	113
	TOT ≈ S1 + S2	7
	TOT calculated by summing scopes since TOT = S1 or S2	5
B	S3 included in total, but TOT = S1 + S2. Both cannot be true.	10
C	S1 exists, S2 missing (3 cities), or in S2 exists, S1 missing (later derived) (6 cities)	9
D	both scopes missing	36
E	S1 exists, S2 missing, and TOT = S1 + S2 = S1. S1 likely correct therefore TOT is incomplete.	7
Emissions quality flags, definitions, and corresponding number of D^CDP^ cities for each. See Figure 3 for more details on the actions taken to obtain each EQF.		

**Table 4:** Gas species breakdown.

Gases included	Number of cities
CO_2_, CH_4_, N_2_O	87
CO_2_	44
CO_2_, CH_4_, N_2_O, HFCs, PFCs, SF_6_	23
CO_2_, CH_4_, N_2_O, HFCs, PFCs, SF_6_, NF_3_	12
CO_2_, CH_4_, N_2_O, HFCs	4
CO_2_, CH_4_	4
CO_2_, CH_4_, SF_6_, N_2_O	2
CO_2_, CH_4_, N_2_O, PFCs	1
CO_2_, CH_4_, SF_6_	1
CO_2_, CH_4_, N_2_O, HFCs, SF_6_	1
CO_2_, CH_4_, N_2_O, PFCs, SF_6_	1
CO_2_, CH_4_, N_2_O, PFCs, HFCs	1
Combination of six gas species (CH_4_, N_2_O, HFCs, PFCs, SF_6_, NF_3_) in addition to CO_2_ included in D^CDP^ and the city count for each combination. Number of cities missing list of gases: 6. Gases included were not available for D^carbonn^.	

**Table 5:** Quality Flag definitions.

Quality Flag	Definition
Emissions Quality Flag (EQF)	Computed for D^CDP^ emissions of each city based on consistency between reported total emissions (TOT and scope-specific values (S1, S2) as shown in the flow-chart of Figure 3
Area Quality Flag (AQF)	Determined from the ratio of city areas across 6 pairs of datasets (X/Y): DCP/GEA, CDP/WB, CDP/OTHERS, PKU/GEA/, PKU/WB, PKU/OTHERS
Population Quality Flag (PQF)	Determined from the absolute difference between the year of Scope-1 emissions in D^emissions^ and the year of population in D^CDP^, D^carbonn^, D^WB^, and D^OTHERS^
Household size Quality Flag (HQF)	Determined from the absolute difference between the year of Scope-1 emissions in D^emissions^ and the year of household size in D^GEA+^and D^OTHERS^
Quality Flags computed for selected attributes and their definition.	
